# Gut microbiome signatures linked to HIV-1 reservoir size and viremia control

**DOI:** 10.1186/s40168-022-01247-6

**Published:** 2022-04-11

**Authors:** Alessandra Borgognone, Marc Noguera-Julian, Bruna Oriol, Laura Noël-Romas, Marta Ruiz-Riol, Yolanda Guillén, Mariona Parera, Maria Casadellà, Clara Duran, Maria C. Puertas, Francesc Català-Moll, Marlon De Leon, Samantha Knodel, Kenzie Birse, Christian Manzardo, José M. Miró, Bonaventura Clotet, Javier Martinez-Picado, José Moltó, Beatriz Mothe, Adam Burgener, Christian Brander, Roger Paredes, the BCN02 Study Group, Susana Benet, Christian Brander, Samandhy Cedeño, Bonaventura Clotet, Pep Coll, Anuska Llano, Javier Martinez-Picado, Marta Marszalek, Sara Morón-López, Beatriz Mothe, Roger Paredes, Maria C. Puertas, Miriam Rosás-Umbert, Marta Ruiz-Riol, Roser Escrig, Silvia Gel, Miriam López, Cristina Miranda, José Moltó, Jose Muñoz, Nuria Perez-Alvarez, Jordi Puig, Boris Revollo, Jessica Toro, Ana María Barriocanal, Cristina Perez-Reche, Magí Farré, Marta Valle, Christian Manzardo, Juan Ambrosioni, Irene Ruiz, Cristina Rovira, Carmen Hurtado, Carmen Ligero, Emma Fernández, Sonsoles Sánchez-Palomino, Jose M. Miró, Antonio Carrillo, Michael Meulbroek, Ferran Pujol, Jorge Saz, Nicola Borthwick, Alison Crook, Edmund G. Wee, Tomáš Hanke

**Affiliations:** 1 IrsiCaixa AIDS Research Institute, Hospital Universitari Germans Trias i Pujol, Barcelona, Catalonia Spain; 2 CIBERINFEC, Madrid, Spain; 3 University of Vic–Central University of Catalonia (UVic–UCC), Vic, Catalonia Spain; 4 Universitat Autonoma de Barcelona (UAB), Barcelona, Catalonia Spain; 5 Center for Global Health and Diseases, Department of Pathology, Case Western Reserve University, Cleveland, OH USA; 6 Department of Obstetrics & Gynecology, University of Manitoba, Winnipeg, Manitoba Canada; 7 Institut Mar d’Investigacions mediques (IMIM), CIBERONC, Barcelona, Catalonia Spain; 8 Infectious Diseases Service, Hospital Clinic-Institut d’Investigacions Biomèdiques August Pi i Sunyer (IDIBAPS), University of Barcelona, Barcelona, Catalonia Spain; 9 Fight AIDS Foundation, Infectious Diseases Department, Germans Trias i Pujol University Hospital, Barcelona, Catalonia Spain; 10 Department of Infectious Diseases Service, Germans Trias i Pujol University Hospital, Barcelona, Catalonia Spain; 11 Catalan Institution for Research and Advanced Studies (ICREA), Barcelona, Catalonia Spain; 12 Department of Medicine Solna, Center for Molecular Medicine, Karolinska Institute, Karolinska University Hospital, Stockholm, Sweden

**Keywords:** HIV-1 post-treatment control, Gut microbiome, Therapeutic HIV-1 vaccine, Viral reservoir size, Microbiome-based predictive biomarker

## Abstract

**Background:**

The potential role of the gut microbiome as a predictor of immune-mediated HIV-1 control in the absence of antiretroviral therapy (ART) is still unknown. In the BCN02 clinical trial, which combined the MVA.HIVconsv immunogen with the latency-reversing agent romidepsin in early-ART treated HIV-1 infected individuals, 23% (3/13) of participants showed sustained low-levels of plasma viremia during 32 weeks of a monitored ART pause (MAP). Here, we present a multi-omics analysis to identify compositional and functional gut microbiome patterns associated with HIV-1 control in the BCN02 trial.

**Results:**

Viremic controllers during the MAP (controllers) exhibited higher *Bacteroidales/Clostridiales* ratio and lower microbial gene richness before vaccination and throughout the study intervention when compared to non-controllers. Longitudinal assessment indicated that the gut microbiome of controllers was enriched in pro-inflammatory bacteria and depleted in butyrate-producing bacteria and methanogenic archaea. Functional profiling also showed that metabolic pathways related to fatty acid and lipid biosynthesis were significantly increased in controllers. Fecal metaproteome analyses confirmed that baseline functional differences were mainly driven by *Clostridiales*. Participants with high baseline *Bacteroidales/Clostridiales* ratio had increased pre-existing immune activation-related transcripts. The *Bacteroidales/Clostridiales* ratio as well as host immune-activation signatures inversely correlated with HIV-1 reservoir size.

**Conclusions:**

The present proof-of-concept study suggests the *Bacteroidales/Clostridiales* ratio as a novel gut microbiome signature associated with HIV-1 reservoir size and immune-mediated viral control after ART interruption.

Video abstract

**Supplementary Information:**

The online version contains supplementary material available at 10.1186/s40168-022-01247-6.

## Background

A major obstacle to HIV-1 cure is the persistence of viral reservoirs. This mainly refers to latently infected cells carrying transcriptionally silent, replication-competent viruses which evade antiretroviral therapy (ART) as well as immune-mediated clearance [1–3]. The immune system is generally unable to contain HIV-1 replication in the absence of ART [4]. However, up to 10–20% of subjects that initiate ART within first weeks after HIV-1 acquisition may temporarily achieve HIV-1 viremia suppression after ART interruption (ATI) [5]. Understanding the mechanisms behind immune-mediated viremia control after ATI is key to progress towards a functional HIV cure. Broader and higher-magnitude CTL (cytotoxic T-lymphocyte) responses against less diverse HIV-1 epitopes [6, 7] in the context of favorable HLA class I genotypes [8] and smaller HIV-1 reservoir size [9] have all been related to such post-treatment HIV-1 control.

There is indirect evidence that the gut microbiome might also contribute to immune-mediated control of HIV-1 replication [10, 11]. Vaccine-induced gut microbiome alterations, consisting in lower bacterial diversity and negative correlation between richness and CD14^+^DR^-^ monocytes in colorectal intraepithelial lymphocytes, have been recently associated with HIV/SIV (SHIV) protection in a non-human primate challenge study after mucosal vaccination with HIV/SIV peptides, modified vaccinia Ankara–SIV and HIV-gp120–CD4 fusion protein plus adjuvants through the oral route [12]. In the HVTN 096 trial [13], where the impact of the gut microbiota on HIV-specific immune response to a DNA-prime, poxvirus-boost strategy in human adults was assessed, baseline and vaccine-induced gp41-reactive IgG titers were associated with different microbiota community structures, in terms of richness and composition [14]. In particular, co-occurring bacterial groups, such as *Ruminococcaceae*, *Peptoniphilaceae*, and *Bacteroidaceae*, were associated with vaccine-induced IgG response and inversely correlated with pre-existing gp41 binding IgG antibodies, suggesting that the microbiome may influence the immune response and vaccine immunogenicity [14]. Also, another study evidence has shown that HIV vaccine-induced CD4+ T and B cell responses could be imprinted by prior exposure to cross-reactive intestinal microbiota-derived antigens [15]. Further evidence emerged from other studies in typhoid Ty21 [16], rotavirus [17] and oral polio virus, tetanus-toxoid, bacillus Calmette-Guérin, and hepatitis B immunization strategies [18], in which specific gut microbiome signatures (*Bifidobacterium*, *Streptococcus bovis,* and *Clostridiales*, respectively) positively correlated with vaccine-induced immune response. In the absence of immune correlates of viral control, HIV cure trials usually incorporate an ART interruption phase to address the efficacy of a therapeutic intervention [19]. Data on the role of gut microbiome composition in the responsiveness to a curative strategy and the relationship with viral control after ART interruption are lacking. The BCN02 study [20] was a single-arm, proof-of-concept “kick and kill” clinical trial evaluating the safety and the in vivo effects of the histone deacetylase inhibitor romidepsin given as a latency reversing agent [21] in combination with a therapeutic HIV vaccine (MVA.HIVconsv) in a group of early-ART treated HIV-1-infected individuals [22, 23]. During a monitored ART interruption (MAP), 23% of individuals showed sustained viremia control up to 32 weeks of follow-up.

Here, we aimed to identify salient compositional and functional gut microbiome patterns associated with control of HIV-1 viremia after ART interruption in the “kick and kill” strategy used in the BCN02 study.

## Materials and methods

### Study design

This was a sub-study derived from the BCN02 clinical trial (NCT02616874). The BCN02 was a multicenter, open-label, single-arm, phase I, proof-of-concept clinical trial in which 15 HIV-1-infected individuals with sustained viral suppression who started ART within the first 6 months after HIV transmission were enrolled to evaluate the safety, tolerability, immunogenicity, and effect on the viral reservoir of a kick and kill strategy consisting of the combination of HIVconsv vaccines with romidepsin, given as a latency reversing agent (LRA) [20] (Additional file 1: Fig. S1a). Romidepsin is a histone deacetylase inhibitor (HDACi), developed as an anti-cancer drug, which has been shown to induce HIV-1 transcription both in vitro and in vivo [21, 24]. The HIVconsv immunogen was constructed by assembling 14 highly conserved regions derived from HIV-1 genes Gag, Pol, Vif, and Env alternating, for each domain, the consensus sequence of the four major HIV-1 clades A, B, C, and D and delivered by non-replicating poxvirus MVA vector [20]. Fifteen individuals enrolled in the BCN02 trial (procedures for recruitment and eligibility criteria are detailed elsewhere [20]) were immunized with a first dose of MVA.HIVconsv (MVA1, 2 × 10^8^ pfu intramuscularly), followed by three weekly-doses of romidepsin (RMD_1-2-3_, 5 mg/m^2^ BSA intravenously) and a second boost of MVA.HIVconsv (MVA2, 2 × 10^8^ pfu intramuscularly) 4 weeks after the last RMD_3_ infusion. To assess the ability for viral control after ART interruption, participants underwent a monitored antiviral pause (MAP), 8 weeks after the second vaccination (MVA2), for a maximum of 32 weeks or until any ART resumption criteria were met (plasma viral load > 2000 copies/ml, CD4^+^ cell counts < 500 cells/mm^3^ and/or development of clinical symptoms related to an acute retroviral syndrome [20]). The study was conducted between February 2016 and October 2017 at two HIV-1 units from university hospitals in Barcelona (Hospital Germans Trias i Pujol and Hospital Clínic) and a community center (BCN-Checkpoint, Barcelona). The microbiome sub-study concept, design, and patient information were reviewed and approved by the institutional ethical review board of the participating institutions (Reference Nr AC-15-108-R) and by the Spanish Regulatory Authorities (EudraCT 2015-002300-84). Written informed consent was provided by all study participants in accordance to the principles expressed in the Declaration of Helsinki and local personal data protection law (LOPD 15/1999).

### Sample disposition and data analysis

Fourteen participants from the BCN02 trial consented to participate in the BCN02-microbiome study, 1 was excluded due to a protocol violation during MAP, and 13 were included for multi-omics analyses. Twelve from the thirteen participants that finalized the “kick and kill” intervention completed the MAP phase (*n* = 3 controllers and *n* = 9 non-controllers) and one subject (B07) did not enter the MAP period due to immune futility pre-defined criteria and absence of protective HLA class I protective alleles associated with natural HIV-1 control (Additional file 1: Fig. S1b). Based on the gut microbiome similarity with non-controllers at study entry and over the “kick and kill” intervention, the participant B07 was included in the non-controller arm to increase the statistical power in this microbiome sub-study. Fecal specimens were longitudinally collected at BCN02 during the intervention period at study entry (pre-Vax), 1 week after 1st vaccination (MVA1), 1 week after RMD_3_ (RMD) and 4 weeks after 2nd vaccination (MVA2). Samples were also collected over the MAP period (from 4 to 34 weeks after ART interruption) and 24 weeks after ART resumption (Additional file 1: Fig. S1a). All samples were processed for shotgun metagenomics analysis. Taxonomical classification, microbial gene content and functional profiling were inferred using Metaphlan2 [25], IGC reference catalog [26], and HUMAnN2 [27], respectively. Sequencing analysis and quality control of metagenomics data are provided in the Additional file 1: Supplementary results. To facilitate the interpretation, longitudinal time points were schematically grouped into three phases (Additional file 1: Fig. S2a). Fecal material, peripheral blood mononuclear cells (PBMC), and plasma samples were also sampled at baseline to assess fecal metaproteome, host transcriptome profiles and soluble inflammation biomarkers, respectively (Additional file 1: Fig. S2b). Microbial proteins from fecal samples were measured by mass spectrometry and protein identification performed using Mascot search engine (v2.4, Matrix Science) and Scaffold Q+ software (v4.9.0, Proteome Software) [28]. PBMC transcriptomes were evaluated using RNA-sequencing and sequence reads aligned to the human reference genome by STAR v2.5.3a [29]. Read counts estimation was inferred using RSEM v1.3.0 [30] and differential expression analysis performed by DESeq2 [31]. Plasma proteins were estimated using the Proximity Extension Assay based on the Olink Inflammation Panel [32]. Correlations between ‘omic’ datasets were computed using Spearman's correlation coefficients and integrative multi-omics analysis was assessed based on the mixOmics R package [33]. A detailed description of wet-lab procedures, bioinformatic methods and statistical analysis of metagenome, metaproteome, transcriptome, soluble plasma markers and multi-omics data is available in the Additional file 1: Supplementary methods.

## Results

### Patient characteristics

In this microbiome sub-study, we evaluated 13 participants of the BCN02 study. Three had sustained low-level HIV plasma viremia (< 2000 copies/ml) during 32 weeks of MAP (viremic controllers), whereas 9 developed HIV-1 RNA rebound (> 2000 copies/ml) during MAP (non-controllers). One additional subject (B07) did not qualify for MAP due to pre-specified immune futility criteria and absence of protective HLA alleles, and therefore, was also considered a non-controller in this microbiome study. (Additional file 1: Fig. S1b). Study participants were predominantly MSM (92%) of Caucasian ethnicity (92%), with median age of 42 years and median body mass index of 22.9 kg/m^2^ (Table 1). Median baseline CD4^+^ T cell counts was 728 (416–1408) cells/mm^3^ and median CD4/CD8 T cell ratio was 1.4 (0.97–1.9). All subjects had been on integrase strand-transfer inhibitor-based triple ART for >3 years, begun during the first 3 months after HIV-1 infection. Median baseline HIV-1 proviral DNA was 140 copies/10^6^ CD4^+^ T cells, being numerically lower in controllers than in non-controllers (65 vs 165 copies/10^6^ CD4^+^ T cells, *p* = 0.29).

**Table 1:** Study participant demographics and clinical characteristics

Variable	All participants (*n* = 13)	Non-controllers (*n* = 10)	Controllers (*n* = 3)
**Demographics**			
Sex (M/F), *n*	12/1	9/1	3/0
Risk group (MSM/HTS), *n*	12/1	9/1	3/0
Ethnic group (Caucasian/Latin), *n*	12/1	9/1	3/0
Age (years)	42 (39–47)	43 (39–47)	34 (33–38)
BMI (kg/m^2^)	22.9 (20.9–24)	22.3 (21.1–23.4)	24.3 (22.2–25)
**Treatment and clinical characteristics**			
ART regimen, *n* (TDF_FTC_RAL/ABC_3TC_RAL/ABC_3TC_DTG)	2/9/2	2/6/2	0/3/0
Viral reservoir (HIV-1 DNA cp/10^6^ CD4^+^ T-cells)	140 (65–361)	165 (76.2–415.7)	65 (62.5–116.5)
CD4^+^ T cell (cells/mm^3^)	728 (648–1182)	839 (581.8–1293.8)	657 (652.5–814)
CD4^+^ T cell (%)	42.9 (42.2–49.3)	43.4 (42.3–48.1)	42.2 (38.4–48.1)
CD4/CD8 T cell counts ratio	1.4 (1.2–1.6)	1.4 (1.2–1.5)	1.3 (1.1–1.6)

Continuous data are presented using median, 25% and 75% interquartile range, unless otherwise described

No statistically significant differences were observed (*p* ≤ 0.05; Wilcoxon rank-sum test)

*M* Male, *F* Female, *MSM* Men who have sex with men, *HTS* Heterosexual, *BMI* Body mass index, *ART* Antiretroviral therapy, *cp* Copies, *TDF* Tenofovir disoproxil fumarate, *FTC* Emtricitabine, *RAL* Raltegravir, *ABC* Abacavir, *3TC* Lamivudine, *DTG* Dolutegravir

### Baseline gut-associated *Bacteroidales/Clostridiales* ratio and lower microbial gene richness discriminate between viremic controllers and non-controllers

**Fig. 1:**
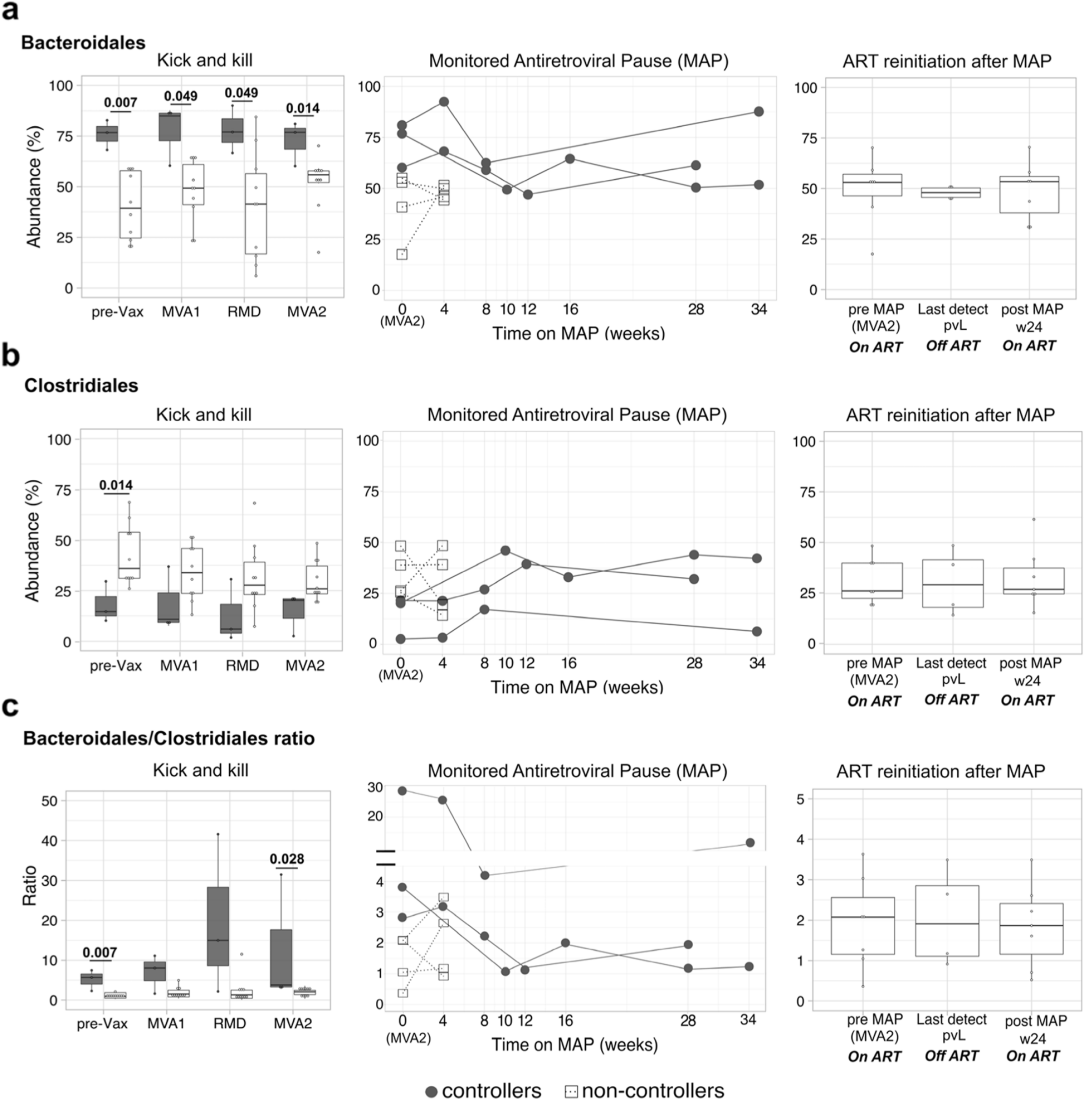
Higher longitudinal *Bacteroidales/Clostridiales* ratio in viremic controllers. Relative abundance expressed as percentage of **a ***Bacteroidales*, **b ***Clostridiales*, and **c** their ratio in controllers (gray) and non-controllers (white) are represented by boxplots (left and right vertical panels) and line plots (middle vertical panels). In line plots, values for each subject are illustrated by white squares (non-controllers) and grey dots (viremic controllers). Boxplots show the median (horizontal black line) and interquartile range between the first and third quartiles (25th and 75th, respectively). Third vertical panels show non-controllers before ART interruption (pre-MAP, *n* = 7), last time point on MAP before ART resumption (last detect pVL, *n* = 4) and 24 weeks after ART resumption (post-MAP w4, *n* = 7). Abbreviations: MAP, monitored antiretroviral pause; pre-Vax, baseline (1 day before first MVA vaccination); MVA1, 1 week after first MVA vaccination; RMD, 1 week after third romidepsin infusion; MVA2, 4 weeks after second MVA vaccination. Unadjusted *p* values are shown. Benjamini–Hochberg multiple hypothesis correction for *p* values ≤ 0.05 are provided in Additional file 2: Dataset S7.

**Fig. 2:**
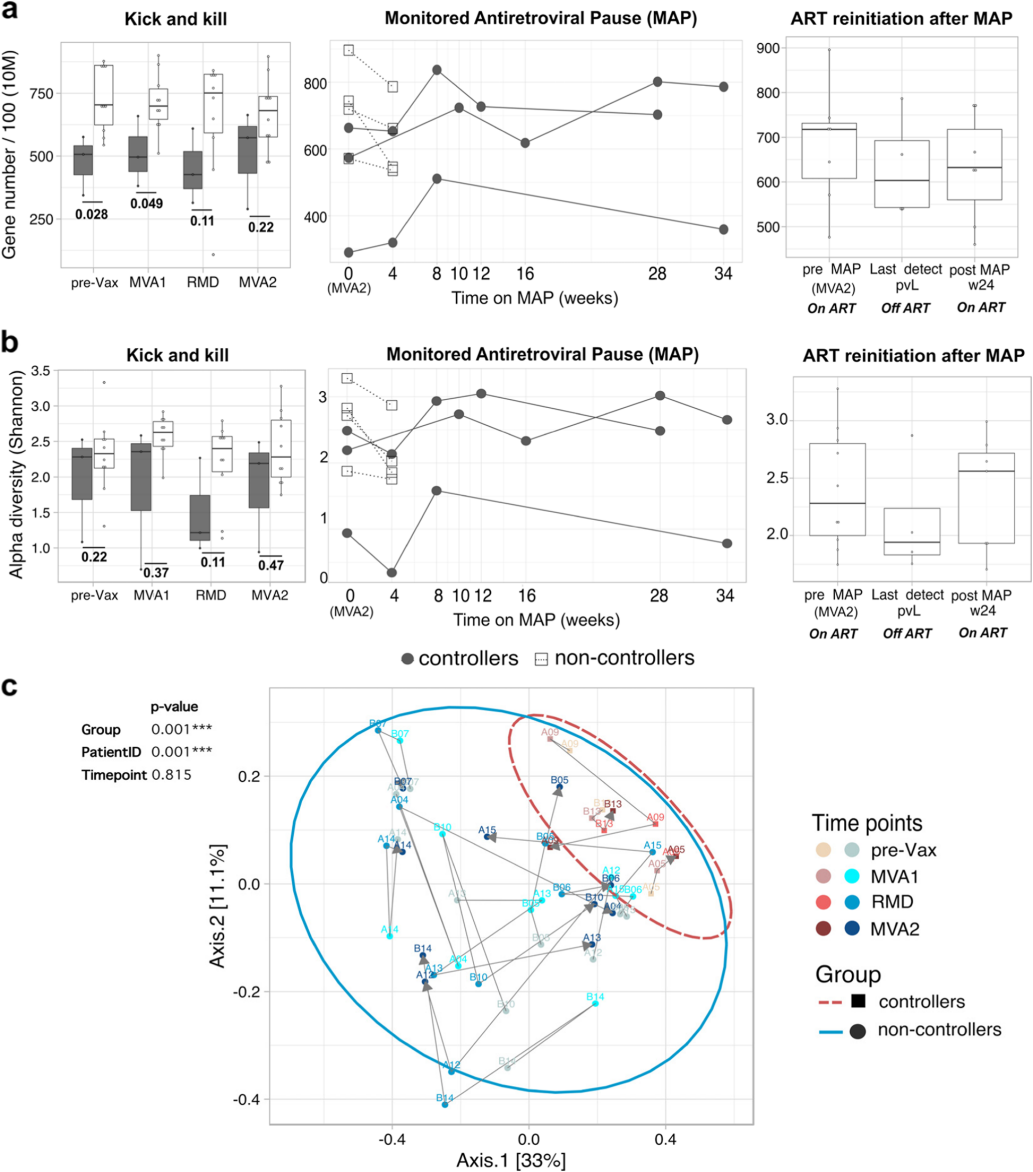
Lower microbial diversity and richness in controllers. Longitudinal **a** microbial gene richness at 10 million (10 M filtered reads) down-sampling size and **b** alpha diversity based on Shannon index in viremic controllers (gray) and non-controllers (white). **c** Principal coordinates analysis (PCoA) of microbial diversity based on Bray-Curtis distances at pre-vaccination and during ‘kick and kill’ intervention. Proportion of variance explained by each principal coordinate axis is reported in the corresponding axis label. Subjects per each group are represented by squares (controllers) and circles (non-controllers). Each point stands for one subject, color coded by group and time point. The increase in purple (controllers) and blue (non-controllers) colors reflects sequential time points from baseline (pre-Vax) to the second vaccine administration (MVA2). Ellipses delineate the distribution of points per each group. Gray arrows link directional changes in bacterial abundance throughout the kick and kill intervention from baseline (pre-Vax). PERMANOVA statistical analysis of samples grouped by group, PatientID (patient internal identifier), and time point is shown on the top of the panel. Abbreviations: MAP, monitored antiretroviral pause; pre-Vax, baseline (1 day before first MVA vaccination); MVA1, 1 week after first MVA vaccination; RMD, 1 week after third romidepsin infusion; MVA2, 4 weeks after second MVA vaccination. Unadjusted *p* values are shown. Benjamini–Hochberg multiple hypothesis correction for *p* values ≤ 0.05 are provided in Additional file 2: Dataset S7

Viremic controllers had significantly higher *Bacteroidales* levels than non-controllers at study entry (pre-Vax *p* = 0.007) and during all the intervention phase (MVA1 *p* = 0.049, RMD *p* = 0.049 and MVA2, *p* = 0.014) (Fig. 1a) as well as lower baseline *Clostridiales* abundance (*p* = 0.014) (Fig. 1b). Accordingly, the *Bacteroidales/Clostridiales* ratio remained significantly higher in controllers at study entry and throughout the intervention (pre-Vax *p* = 0.007 and MVA2, *p* = 0.028) (Fig. 1c). Also, controllers were significantly depleted in archaeal members from the methanogenic order *Methanobacteriales* (Additional file 1: Fig. S4). More detailed analyses at lower taxonomic level within the orders *Bacteroidales* and *Clostridiales* showed that controllers were mainly depleted in *Clostridiales* species, such as *Eubacterium* spp. and *Subdoligranulum* spp., whereas the *Bacteroidales* species *Prevotella copri* was significantly higher after the three romidepsin doses (Additional file 1: Fig. S5), being such trends maintained throughout the intervention (Additional file 1: Figs. S5 and S6). Viremic controllers also had lower microbial gene richness than non-controllers at the study entry (*p* = 0.028) and MVA1 (*p* = 0.049), although such differences lost statistical significance in the RMD and MVA2 assessments (Fig. 2a). Alpha diversity also remained numerically lower in controllers, but differences were not statistically significant (Fig. 2b). Controllers exhibited lower beta-diversity, particularly already at the study entry (*p* = 0.004; Additional file 1: Fig. S7), and showed less intra-host longitudinal evolution than non-controllers (*p* = 0.001) (Fig. 2c). Whereas the gut microbiome composition of controllers was significantly different from that of non-controllers (*p* = 0.001), no significant longitudinal differences were observed across time points (*p* = 0.815), suggesting that the combined intervention did not significantly alter the gut-microbiome composition (Fig. 2c). Of note, results did not change after removing B07 from the non-controller arm (Additional file 1: Fig. S8). No differences in *Bacteroidales* and *Clostridiales* abundances and microbial diversity were observed during MAP and after ART re-initiation (Additional file 1: Supplementary results). Furthermore, longitudinal profiling of metabolic pathways associated with *Bacteroidales*, *Clostridiales*, and archaea showed that controllers were mainly enriched in functions related to fatty acid biosynthesis, whereas functions related to methanogenesis and carbohydrate biosynthesis were overrepresented in non-controllers (Additional file 1: Supplementary results and Figs. S9–S12). Taken together, these data showed that differences between controllers and non-controllers mainly emerged from resident microbial communities, before any intervention was started in BCN02 study. Thus, subsequent analyses were focused on characterizing further discriminant signatures at study entry.

### Increased *Bacteroidales/Clostridiales* ratio in viremic controllers negatively correlated with longitudinal HIV-1 viral reservoir size

**Fig. 3:**
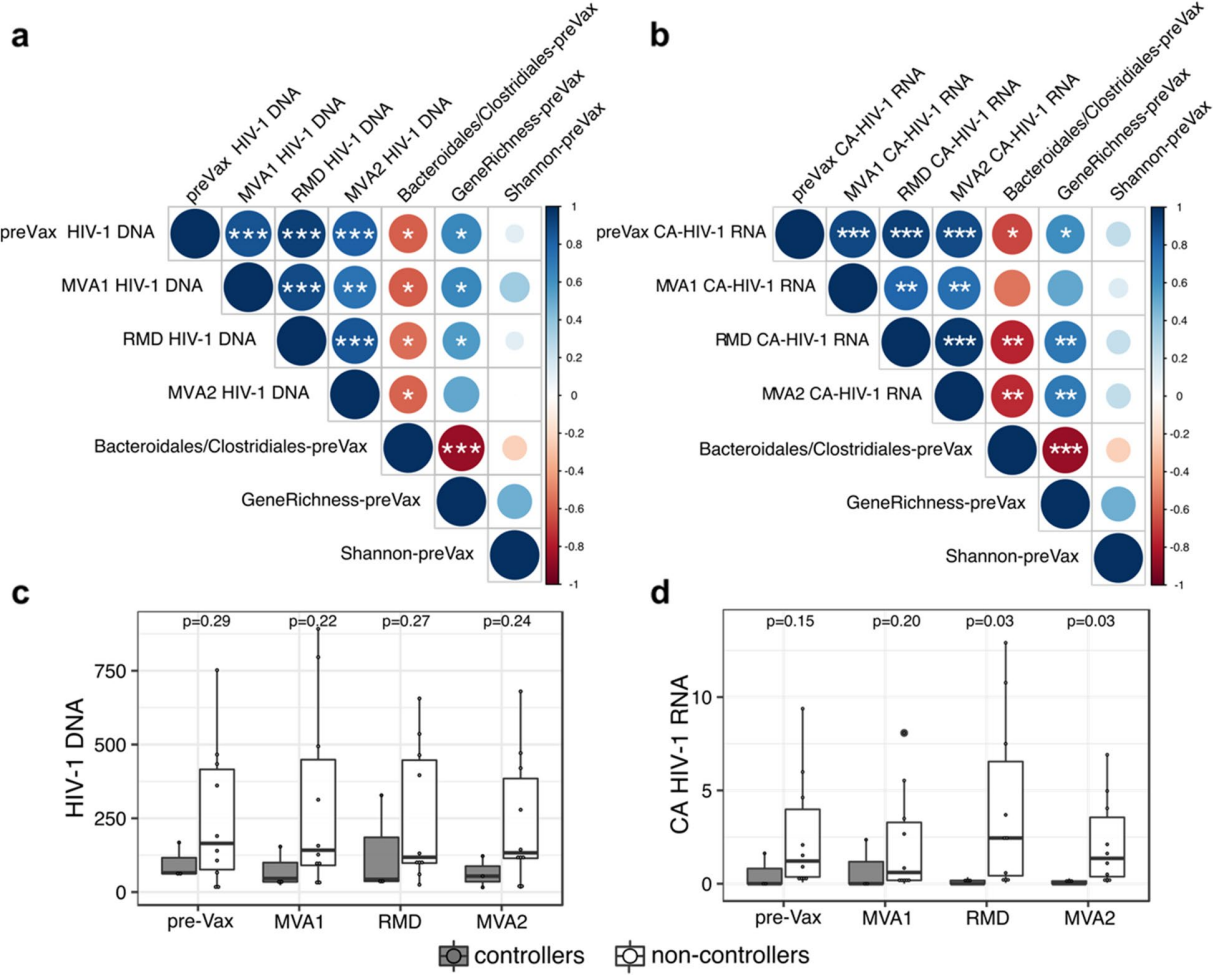
Associations between HIV-1 reservoir size and gut microbial signatures. Spearman’s correlations between gut microbial signatures (ratio *Bacteroidales/Clostridiales*, gene richness, and alpha-diversity Shannon index) and longitudinal **a** HIV-1 DNA (HIV-1 DNA copies/10^6^ CD4^+^ T cells) and **b** cell-associated (CA) HIV-1 RNA (HIV-1/TBP relative expression). Positive correlations are indicated in blue and negative correlations, in red. Color and size of the circles indicate the magnitude of the correlation. White asterisks indicate significant correlations (*p < 0.05; **p < 0.01; ***p < 0.001, Benjamini–Hochberg adjustment for multiple comparisons). Boxplots showing longitudinal comparison of **c** HIV-1 DNA and **d** CA HIV-1 RNA between controllers and non-controllers. Abbreviations: MAP, monitored antiretroviral pause; pre-Vax, baseline (1 day before first MVA vaccination); MVA1, 1 week after first MVA vaccination; RMD, 1 week after third romidepsin infusion; MVA2, 4 weeks after second MVA vaccination

The *Bacteroidales/Clostridiales* ratio inversely and significantly correlated with longitudinal total CD4^+^ T cell-associated HIV-1 DNA measured at study entry (rho  = − 0.6, *p*_*adj*_  =  0.03) and over the intervention, whereas an opposite trend was observed for gene richness (rho = 0.65, *p*_*adj*_  =  0.01 at study entry) (Fig. 3a). A similar trend was observed for cell-associated (CA) HIV-1 RNA (*Bacteroidales/Clostridiales;* rho  = − 0.7, *p*_*adj*_   = 0.01 and gene richness; rho  = 0.61, *p*_*adj*_  =  0.0 at study entry), although stronger correlations were found at RMD and MVA2 (Fig. 3b). In both assessments, alpha-diversity (Shannon index) exhibited weak positive correlation with the viral reservoir, being correlations not significant. Moreover, baseline ratio *Bacteroidales/Clostridiales* and gene richness showed a strong negative correlation (r ho = − 0.87, *p*_*adj*_  =  0.0001) (Fig. 3a, b), in line with trends observed in the microbiota characterization. In the longitudinal comparison, controllers tended to displayed lower viral reservoir size (Fig. 3c, d), although differences were statistically significant only for CA HIV-1 RNA at RMD and MVA2 (*p* = 0.03) (Fig. 3d). An additional set of clinical and vaccine-response variables was screened for association with gut microbial signatures. Absolute CD4^+^ T cell count before ART initiation was the only factor significantly associated with the *Bacteroidales/Clostridiales* ratio (rho = 0.65, *p*_*adj*_  =  0.01) and gene richness (r ho = − 0.62, *p*_*adj*_  =  0.02), whereas a strong and inverse correlation was found between the Shannon index and CD4/CD8 ratio at BCN02 study entry (rho = 0.9, *p*_*adj*_ =  2.83e−05) (Additional file 1: Fig. S13).

### Distinct bacterial protein signatures associated with viremia control

**Fig. 4:**
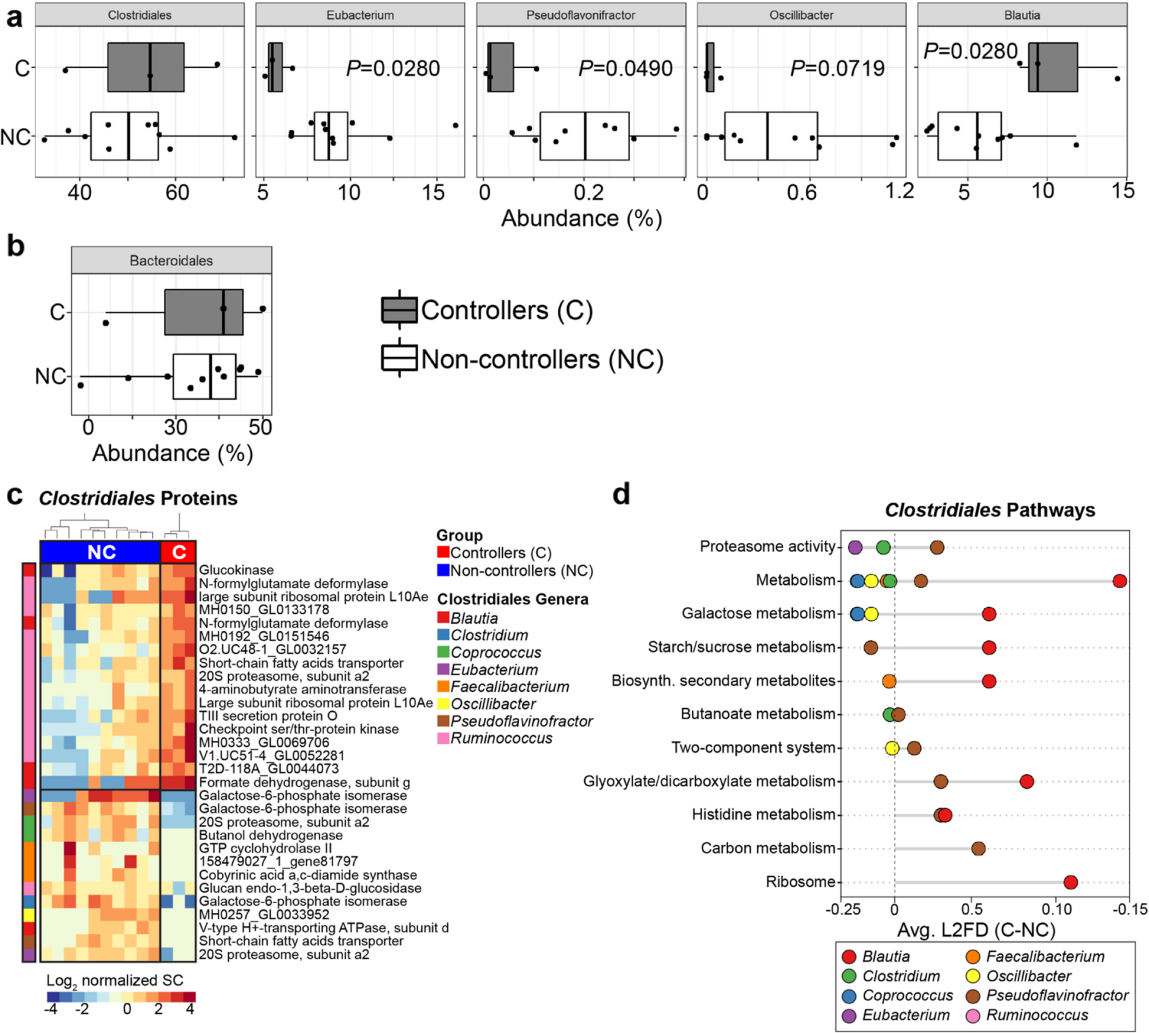
Baseline metaproteomic signatures associated with HIV control after ART interruption. Comparison of **a ***Clostridiales* and **b ***Bacteroidales* abundances derived from gut metaproteome data in controllers and non-controllers at study entry. No differences in *Clostridiales* or *Bacteroidales* proteome at the order-level were observed. **c** Baseline levels of *Clostridiales* proteins distinguished controllers from non-controllers in a hierarchical clustering. **d** Functional annotation of *Clostridiales* bacterial proteins using KEGG gene ontology identified baseline differences in cellular metabolism pathways between groups. Abbreviations: C = controllers, NC = non-controllers, SC = spectral count. Unadjusted *p* values are shown. Benjamini–Hochberg multiple hypothesis correction for *p* values ≤ 0.05 are provided in Additional file 2: Dataset S7

Using baseline proteomic data (15,214 bacterial proteins in total), 24 orders and 69 genera were quantified across samples (Additional file 2: Dataset S2). The abundance of total *Clostridiales* and *Bacteroidales* orders derived from proteome data was not different between groups (Fig. 4a, b). However, several *Clostridiales* genera were decreased in controllers, i.e., *Eubacterium* (− 3.71%; *p* = 0.03), *Pseudoflavonifractor* (− 0.49%; *p* = 0.049), *Oscillibacter* (− 0.14%; *p* = 0.07), whereas *Blautia* was increased (+ 5.02%; *p* = 0.03) (Fig. 4a). Unbiased hierarchical clustering based on *Clostridiales* genera showed protein differences (*p* < 0.025) between groups (Fig. 4c). Viremic controllers were enriched in bacterial proteins from *Blautia* and *Ruminococcus*, and depleted in proteins derived from other *Clostridiales* such as *Clostridium*, *Eubacterium*, *Coprococcus*, *Faecalibacterium*, *Oscillibacter*, and *Pseudoflavinofactor*. Pathways associated with *Blautia* included galactose, starch/sucrose, and glyoxylate/dicarboxylate metabolism as well as ribosome activity (Fig. 4d). Butyrate and other short-chain fatty acid metabolism pathways were similar in both groups (Fig. 4c, d).

### Increased baseline immune activation and inflammatory response transcripts in viremic controllers

**Fig. 5:**
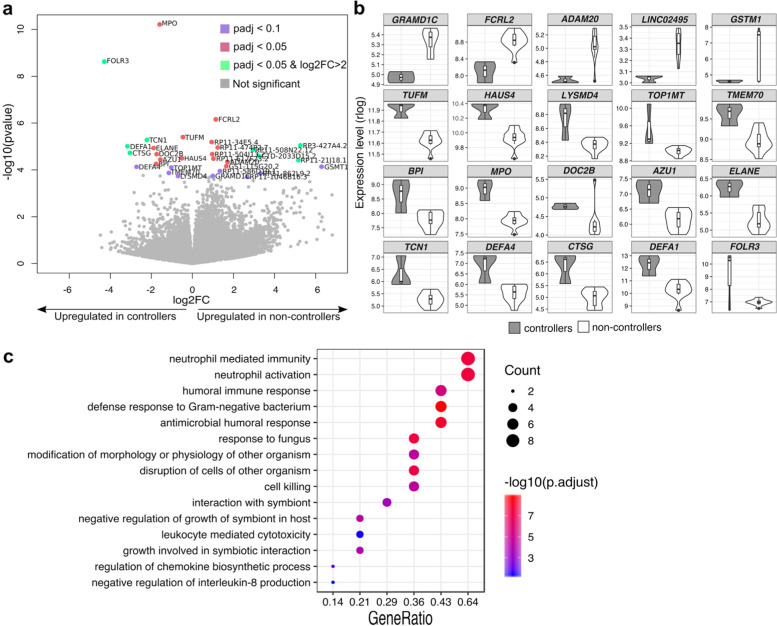
Baseline functional enrichment in levels of immune activation and inflammatory response in viremic controllers. **a** Volcano plot of differentially expressed genes between controllers and non-controllers at baseline (pre-Vax) with adjusted *p* value < 0.1 (violet dots), adjusted *p* value < 0.05 (red dots) and log2 (foldchange) > 2 and adjusted *p* value < 0.05 (green dots). Gray-colored dots represent genes not displaying statistical significance (BH-adjusted *p* value > 0.1). The log2 fold change on the *x*-axis indicates the magnitude of change, while the −log10 (*p*-adjust) on the *y*-axis indicates the significance of each gene. **b** Violin plots showing relative expression levels (rlog, regularized log transformation) of differentially expressed genes with functional annotation. **c** Gene ontology (GO) enrichment analysis of upregulated genes in controllers. In the *y*-axis, only representative enriched GO terms (biological process) are reported (terms obtained after redundancy reduction by REVIGO). *X*-axis reports the percentage of genes in a given GO terms, expressed as ‘Gene ratio’. The color key from blue to red indicates low to high Bonferroni-adjusted log 10 *p* value. Dot sizes are based on the 'count' (genes) associated to each GO term. Significantly enriched GO terms, number of genes associated with each GO term and adjusted *p* values are provided in Additional file 3: Table S3

Full-PBMC gene expression analysis detected a total of 27,426 transcripts at baseline, after filtering for low-expressed genes (Additional file 2: Dataset S3). Using DESeq2 [30], a total of 31 differentially expressed genes (DEGs) were identified (log_2_ FoldChange = 0 and *p*_*adj*_ < 0.1), of which 15 and 16 were upregulated in controllers and non-controllers, respectively (Fig. 5a and Additional file 3: Table S1). Hierarchical clustering based on transcriptional DEG profiles showed that controllers grouped together, while non-controllers separated into two distinct expression groups (Additional file 1: Fig. S14a). Upregulated genes in non-controllers included 11 transcripts with unknown function (Additional file 3: Table S2), which were excluded from downstream analyses. Upregulated genes in controllers (Fig. 5b and Additional file 1: Fig. S14b-c), such as myeloperoxidase (*MPO)*, defensin alpha 1 and 4 (*DEFA1, DEFA4*), and neutrophil elastase (*ELANE*) (Additional file 3: Table S2) were known to be implicated in immune response signaling and regulation of inflammatory processes [34, 35]. Gene Ontology (GO) analysis confirmed that genes upregulated in controllers were enriched in functions related to immune system activation, such as neutrophil-mediate immunity, leukocyte degranulation and antimicrobial humoral response (Fig. 5c and Additional file 3: Table S3). Moreover, a group of inflammation-related plasma proteins was significantly increased in controllers at baseline (Additional file 1: Supplementary results and Fig. S15).

### Integration analysis between *Bacteroidales/Clostridiales* ratio, host immune activation transcripts, bacterial proteins, and HIV-1 reservoir size

**Fig. 6:**
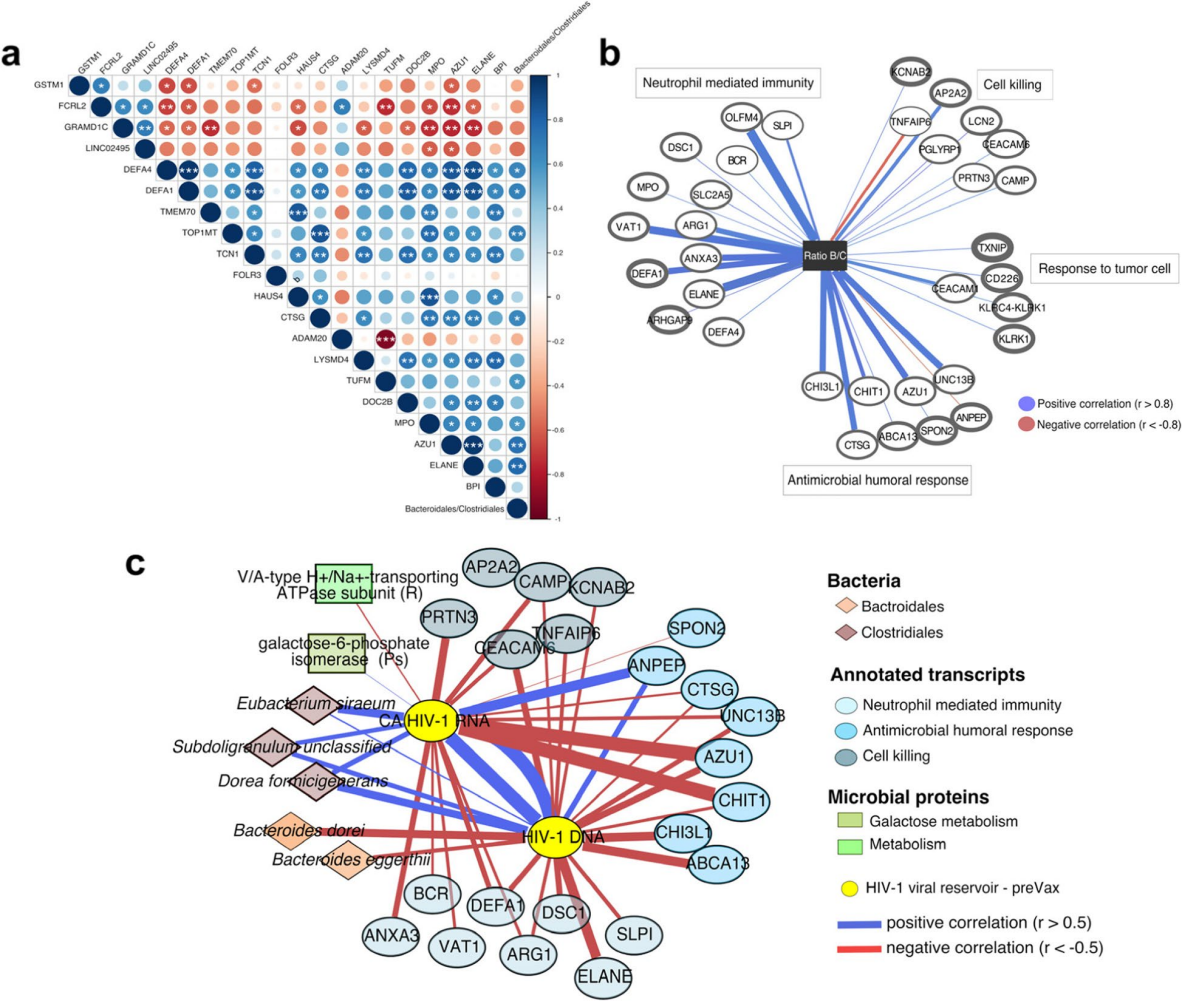
Integrative analysis of baseline gut microbial signatures, immune activation-related transcripts, bacterial proteins and HIV-1 reservoir. **a** Spearman’s correlations between the ratio *Bacteroidales/Clostridiales* and DEGs (annotated transcripts). Color and size of the circles indicate the magnitude of the correlation. White asterisks indicate significant correlations (*p < 0.05; **p < 0.01; ***p < 0.001, Benjamini–Hochberg adjustment for multiple comparisons). **b** Network visualizing significant Spearman’s correlations between the ratio *Bacteroidales/Clostridiales* and transcripts involved in the enrichment analysis described in Additional file 3: Table S4. Transcripts are represented as vertices and border width is proportional to transcript expression (log2 |cpmTMM_w0 +1|) in controllers. Edge width indicates the magnitude of correlation. **c** Network showing Spearman's correlation between viral reservoir (CA HIV-1 RNA and HIV-1 DNA), bacterial species within *Bacteroidales* and *Clostrdiales*, host transcripts correlated with the ratio *Bacteroidales/Clostrdiales* and bacterial proteins (*p* ≤ 0.025). Features are showed as vertices and colored by ‘omic’ dataset. Edge width indicates the magnitude of correlation coefficient. Protein-associated bacterial genera are reported in parentheses. In all panels, positive and negative correlations are indicated in blue and red, respectively. Abbreviations: DEGs, differentially expressed genes between controllers and non-controllers; R, *Ruminococcus*; Ps, *Pseudoflavonifactor* and pre-Vax, baseline timepoint (1 day before first MVA vaccination)

The baseline *Bacteroidales/Clostridiales* ratio positively correlated (*p*_*adj*_  < 0.05) with differentially expressed genes involved in inflammatory response and immune system activation, including *DEFA1*, *DEFA4*, *TOP1MT*, *CTSG*, *MPO*, *AZU1*, *ELANE* (Fig. 6a, Spearman rho and adjusted *p* values are given in Additional file 2: Dataset S5). Additional correlation and enrichment analysis extended to the full set of host transcripts supported such observation (Fig. 6b), also showing significant associations with the baseline viral reservoir (Additional file 1: Supplementary results and Fig. S16). In the integrated analysis of metagenomic, transcriptomic, and metaproteomic data for the identification of discriminating signatures between controllers and non-controllers, *Bacteroidales* and *Clostridiales* were clearly separated through the components (Additional file 1: Fig. S17a). While *Bacteroidales* clustered and positively associated with immune activation transcripts (*MPO*, *AZU1*, *ELANE*, *TCN1*, *DEFA1*, *BPI*, *DEF4*) as well as proteins from *Ruminococcus*, *Blautia*, and *Prevotella*, the order *Clostridiales* inversely correlated with such features (Additional file 1: Fig. S17a-b). Multi-omics correlations at lower taxonomic scale, including viral reservoir data confirmed that *Bacteroidales* species (*B. dorei* and *B. eggerthii*) inversely correlated with HIV-1 DNA levels, whereas members of *Clostridiales* (*S. unclassified*, *D. formicigenerans*, and *E. siraeum*) positively correlated with both HIV-1 DNA and CA HIV-1 RNA (Fig. 6c). In turn, viral reservoir size negatively correlated with genes involved in neutrophil-mediated immunity and host defense (Fig. 6c and Additional file 2: Dataset S6). These data together showed positive associations between *Bacteroidales* taxa and host transcripts related to immune system activation, and in turn negative correlation with the HIV-1 viral reservoir. Whereas, members within the order *Clostridiales* showed the opposite trend. Such observations emerged by analyzing either the ratio or individual taxa, further supporting potential pre-existing interactions between intestinal *Bacteroidales* species, host immune activation, and reservoir size in viremic controllers.

## Discussion

In this proof-of-concept study, a longitudinal multi-‘omics’ analysis identified the *Bacteroidales/Clostridiales* ratio as a novel gut microbiome signature associated with HIV-1 reservoir size and viremic control during a monitored ART pause. Individuals with high *Bacteroidales/Clostridiales* ratio showed gene expression signatures related to immune activation, particularly neutrophil-mediated immunity and antimicrobial humoral response, which negatively correlated with the viral reservoir size. Our findings largely arise from unsupervised analyses where many other signatures could have emerged, especially given the relatively low number of individuals analyzed. However, they are internally coherent and consistent with a theoretical framework where increased inflammation might contribute to immune-mediated HIV-1 control. They also suggest a putative biomarker for safer ART interruptions in HIV cure studies.

The gut microbiome of controllers was enriched in pro-inflammatory species, such as *P. copri* [36], and depleted in bacteria, traditionally associated with the maintenance of gut homeostasis through production of SCFAs [37], including *R. intestinalis* and *Subdoligranulum spp*. Lower microbial diversity and gene richness found in controllers were consistent with a previous work from our group in people living with HIV [38], as well as other studies [39], in which higher gene richness associated with increased levels of butyrate-producing bacteria and methanogenic archaea. Microbial functional enrichment in lipid and fatty acid biosynthesis in controllers might be reflective of mechanisms of lipopolysaccharide biosynthesis and production of inflammatory mediators [40] mediated by members of *Bacteroidales* [41]. No discernible longitudinal variations were observed in the gut microbiome of BCN02 participants, in line with previous evidence in oral typhoid immunization [16]. Of note, the gut microbiome in healthy population has been described generally resilient to perturbations [42]. Taken together, these observations would suggest a trend toward the maintenance of a relative stability in the gut microbiome composition upon vaccination. Baseline metaproteome analysis confirmed that functional differences between controllers and non-controllers were mainly driven by *Clostridiales*, which were actively producing microbial proteins in both groups albeit in distinct functional contexts. Further discriminating baseline signatures linked to increased immune system activation and inflammatory response in controllers emerged from PBMC transcriptome and inflammation-related plasma proteins profiling. It also emerged that the ratio *Bacteroidales/Clostridiales* inversely correlated with the viral reservoir size in terms of HIV-1 DNA and CA HIV-1 RNA. Although controllers did not display significantly lower viral reservoir size compared to non-controllers, such associations were consistent with previous studies suggesting a role of low viral reservoir on ART interruption outcomes [9]. The results of our independent analyses (i.e., different omics approaches), as well as their integration suggest that baseline immune activation potentially associated with a microbial shift toward pro-inflammatory bacteria and lower viral reservoir may contribute to sustained post-ART interruption HIV-1 control. While there is evidence suggesting a strong impact of the gut microbiota composition on host immune system and inflammatory status [43], the mechanistic basis of how microbial communities may interact with the viral reservoir and, in turn, exert immunomodulatory effects on HIV-1 control during ART interruption remains to be delineated. We speculate that a pre-existing, altered balance of ‘beneficial’ gut microbial groups, such as *Clostridiales*, and concomitant overabundance of pro-inflammatory bacteria would boost host immune system activation, thus triggering a prompt control of rebounding virus, as observed in controllers. In support to this hypothesis, increased abundance of members from *Clostridiales* were previously associated with neutrophilia and lower poliovirus and tetanus-hepatitis B vaccine response [18]. Moreover, baseline transcriptional pro-inflammatory and immune activation signatures were suggested as potential predictors of increased influenza [44], systemic lupus erythematosus [45] and hepatitis B [46] vaccine-induced immune response, with weaker responses in elderly [44, 46]. It is then reasonable to postulate that immune activation prior vaccination together with microbiome-associated factors may affect vaccine outcomes. Results obtained in this exploratory study are indented to generate hypotheses and potentially contribute to establish a coherent framework for subsequent confirmatory studies. In this context, several studies have suggested plausible mechanisms by which the microbiota could modulate immune responses to vaccination in humans [47]). Despite causal links remain to be fully deciphered, the ability of the microbiota to directly interact with immune cells in the gut as well as to regulate the systemic availability of critical metabolites in distal locations is well-established [47]. A few studies also attempted to investigate the exact role played by the gut microbiota in HIV control. Despite gut dysbiosis persistence in HIV-1 infected patients after ART initiation (reduced gut microbial richness and depletion in anti-inflammatory bacteria [48]), microbial richness may be restored by prolonged ART treatment [49]. Also, elite controllers, who spontaneously control HIV replication without ART, showed higher gut microbial richness with a metabolic profile resembling that of HIV-uninfected adults [50] and increased Th17 cells in the gut mucosa compared to ART-treated patients [51]. In line with our results, a recent study showed enriched levels of Bacteroidetes genera, such as *Bacteroides* and *Prevotella*, in patients with sustained HIV control during ART interruption, after receiving dendritic cells-based HIV-1 immunization [52]. Moreover, additional evidence on HIV virologic controllers receiving a TLR7 agonist before ART interruption with baseline enrichment in *P. copri* and negative association between abundance of *Ruminococcus gnavus* and time to viral rebound suggested a potential impact of certain microbiome species on HIV persistence [53]. Together, such observations illustrate the exceptional challenge in deconvoluting the complex dynamics between specific microbial species or byproducts and HIV-1 control. Our study has several limitations. Due to eligibility criteria in the parental BCN02 study, there was a limited sample size, and we were unable to include a control arm without the intervention. Our considerations were therefore narrowed to three individuals showing viremic control during ART interruption. In addition, given the small sample size and limitations of non-parametric methods coupled with multiple testing correction [54], we used a reasonable threshold of 10% false-discovery rate (adjusted *p* value < 0.1) to prevent false negatives. Another fundamental issue to consider is that this sub-study was largely restricted to Caucasian/MSM population. Large-scale studies have revealed broad differences in the gut microbiome composition [55] and modes of HIV transmission [56] across distinct geographical populations, thus leading to dramatic variations in the characteristics of study participants at study entry [57]. MSM sexual behavior itself was associated with *Prevotella*-rich/*Bacteroides*-poor microbiota profile and increased microbial diversity [58], suggesting that failure to control for this factor could confound data interpretation. Yet, most of the HIV-associated microbiome studies published to date, including this, have largely focused on single populations or individuals [57], posing the question on the generalizability of the results in different population settings. Ensuring diversity among study participants (i.e., in ethnic group, sexual behavior, or transmission mode) in future microbiome studies, is then critical to both identify population-specific features and to achieve broader-reaching biological conclusions. Bearing these limitations in mind, our results should be interpreted with caution, emphasizing the need of independent validation in randomized and placebo-controlled trials to assess potentially unmeasured confounders and provide further perspectives on factors that might induce gut microbial shifts. Upcoming analyses in larger longitudinal trials, including the recently reported AELIX-002 trial [59], where fecal samples have been stored longitudinally, are expected to validate our results. These preliminary findings might have important implications in the design of HIV-1 cure intervention trials that include ART interruption. As proposed for other therapeutic areas [60], microbiome-associated predictive patterns could help to optimize patient stratification, thus resulting in more targeted studies and higher efficacy of HIV-1 interventions. In addition, if a given resident microbial community is to be defined that is indeed predictive of viral control during ART interruption, then modulating participants’ gut microbiota before immunization might potentially impact vaccine responsiveness and ultimately, clinical outcomes. While host-genetics and other vaccine-associated factors as baseline predictors are less amendable, the gut microbiome is potentially modifiable and even transferrable to another host. Strategies manipulating the gut microbiota composition and relative by-products via prebiotics and/or probiotics administration [61] or microbiota engraftment following fecal microbiota transplantation [62] are under intense evaluation [63], albeit with several limitations.

## Conclusions

In this exploratory study, we identified pre-existing gut microbial and immune activation signatures as potential predictors of sustained HIV-1 control in the absence of ART, providing a potential target for future treatment strategies and opening up new avenues for a functional HIV cure.

## Supplementary Information

**Additional file 1: **Supplementary methods. Supplementary results. **Figure S1.** BCN02-microbiome study design and sample collection strategy. **Figure S2.** Overview of sample disposition for multi-omic analysis. **Figure S3.** Taxonomic classification of fecal samples at the order level. **Figure S4.** Differential abundance in *Methanobacteriales* between controllers and non-controllers. **Figure S5.** Linear Discriminant Analysis (LDA) effect size (LEfSe) at the species level. **Figure S6.** Longitudinal feature-volatility analysis of *Bacteroidales* and *Clostridiales* species. **Figure S7.** Bray-Curtis dissimilarity index between controllers and non-controllers. **Figure S8.** Gut microbiome profiling excluding B07 participant from non-controllers arm. **Figure S9.** Differentially abundant metabolic pathways from *Bacteroidales* and *Clostridiales* at the study entry. **Figure S10.** Differential metabolic pathways between controllers and non-controllers. **Figure S11.** Longitudinal variation of differentially abundant pathways over the trial. **Figure S12.** Differential archaeal metabolic pathways between controllers and non-controllers. **Figure S13.** Spearman’s correlation between clinical data, vaccine response and gut microbial variables. **Figure S14.** Differentially expressed PBMC host genes between controllers and non-controllers at baseline. **Figure S15.** Protein inflammation markers from controllers and non-controllers at baseline. **Figure S16.** Functional enrichment of transcripts correlated with the ratio *Bacteroidales:Clostridiales* and viral reservoir. **Figure S17.** Integrated analysis of microbiome, metaproteome and transcriptome data.

**Additional file 2: Dataset S1.** Longitudinal microbial composition (relative abundance) in viremic controllers and non-controllers analyzed by MetaPhlAn2.0. **Dataset S2.** Raw bacterial protein counts at baseline in viremic controllers and non-controllers analysed using Scaffold Q+ software v4.9.0. **Dataset S3.** Raw gene counts at baseline in viremic controllers and non-controllers estimated using RSEM v1.3.0. **Dataset S4.** Soluble factors (normalized protein expression units) from the Olink inflammation panel at baseline in viremic controllers and non-controllers. **Dataset S5.** List of transcripts significantly correlated with the Bacteroidales:Clostridiales ratio adjusted for multiple comparisons (spearman’s rho and corresponding q-value<0.05 are reported). **Dataset S6.** Spearman’s correlation (rho=0.5) between viral reservoir (CA HIV-1 RNA and HIV-1 DNA), bacterial species (Bacteroidales and Clostrdiales), transcripts correlated with the ratio Bacteroidales / Clostridiales and differentially abundant microbial proteins (*p*<0.025). **Dataset S7.** Benjamini–Hochberg multiple hypothesis correction for unadjusted *p*-values ≤ 0.05 (metagenomics and metaproteomics datasets).

**Additional file 3: Table S1.** List of differentially expressed genes between controllers and non-controllers at baseline (adjusted *p*-value <0.1and log2 FoldChange = 0). **Table S2.** Detailed information of differentially expressed genes (DEGs) between controllers and non-controllers at baseline (adjusted *p*-value <0.1 and log2 FoldChange = 0). **Table S3.** GO terms from enrichment analysis (biological process) based on upregulated genes in viremic controllers (n. transcripts =15). **Table S4.** Enriched GO biological process terms of host transcripts significantly correlated with the ratio Bacteroidales/Clostridiales. **Table S5.** Enriched GO biological process terms of transcripts (n=453) significantly correlated with the ratio Bacteroidales/Clostridiales and viral reservoir (CA HIV-1 RNA and HIV-1 DNA). **Table S6.** Summary of shotgun metagenomics sequencing yield from longitudinally-collected fecal samples. Statistics of reads mapped to integrated gene catalog (IGC) are also shown.

## Data Availability

Datasets supporting the conclusions of this study are available as additional information (Additional file 2). Metagenome and RNA-seq data have been deposited in the European Nucleotide Archive (ENA) and are accessible through ENA accession numbers PRJEB42384 and PRJEB43195. The code and databases used for data analysis are available at 10.5281/zenodo.4876340

## Abbreviations

ART: Antiretroviral therapy
ATI: Antiretroviral therapy interruption
BH-adjusted: Benjamini-Hochberg-adjusted
HUMAnN2: HMP unified metabolic analysis network v.2
MAP: Monitored antiretroviral therapy pause
MVA: MVA.HIVconsv
RMD: Romidepsin
SCFA: Short chain fatty acids
