# Supplementary material for: Gut microbiome signatures linked to HIV-1 reservoir size and viremia control
Source: Microbiome. 2022 Apr 11;10:59. doi: 10.1186/s40168-022-01247-6 (PMC9004083; doi:10.1186/s40168-022-01247-6)
Supplement: Supplementary file 2 — Additional file 1: Supplementary methods. Supplementary results. Figure S1. BCN02-microbiome study design and sample collection strategy. Figure S2. Overview of sample disposition for multi-omic analysis. Figure S3. Taxonomic classification of fecal samples at the order level. Figure S4. Differential abundance in Methanobacteriales between controllers and non-controllers. Figure S5. Linear Discriminant Analysis (LDA) effect size (LEfSe) at the species level. Figure S6. Longitudinal feature-volatility analysis of Bacteroidales and Clostridiales species. Figure S7. Bray-Curtis dissimilarity index between controllers and non-controllers. Figure S8. Gut microbiome profiling excluding B07 participant from non-controllers arm. Figure S9. Differentially abundant metabolic pathways from Bacteroidales and Clostridiales at the study entry. Figure S10. Differential metabolic pathways between controllers and non-controllers. Figure S11. Longitudinal variation of differentially abundant pathways over the trial. Figure S12. Differential archaeal metabolic pathways between controllers and non-controllers. Figure S13. Spearman’s correlation between clinical data, vaccine response and gut microbial variables. Figure S14. Differentially expressed PBMC host genes between controllers and non-controllers at baseline. Figure S15. Protein inflammation markers from controllers and non-controllers at baseline. Figure S16. Functional enrichment of transcripts correlated with the ratio Bacteroidales:Clostridiales and viral reservoir. Figure S17. Integrated analysis of microbiome, metaproteome and transcriptome data. [file 40168_2022_1247_MOESM2_ESM.docx]

**Additional File for:**

**Gut Microbiome Signatures Linked to HIV-1 Reservoir Size and Viremia Control**

Alessandra Borgognone^1*^, Marc Noguera-Julian^1,2,3^, Bruna Oriol^1,4^, Laura Noël-Romas^5,6^, Marta Ruiz-Riol^1,2^, Yolanda Guillén^7^, Mariona Parera^1^, Maria Casadellà^1^, Clara Duran^1,4^, Maria C. Puertas^1,2^, Francesc Català-Moll^1^, Marlon De Leon^5^, Samantha Knodel^5,6^, Kenzie Birse^5,6^, Christian Manzardo^8^, José M. Miró^2,8^, Bonaventura Clotet^1,2,3,4,9,10^, Javier Martinez-Picado^1,2,3,11^, José Moltó^2,9,10^, Beatriz Mothe^1,2,3,9,10^, Adam Burgener^5,6,12^, Christian Brander^1,2,3,11^, Roger Paredes^1,2,3,4,5,9,10*^ & the BCN02 Study Group

^1^ IrsiCaixa AIDS Research Institute, Hospital Universitari Germans Trias i Pujol, Barcelona, Catalonia, Spain

^2^ CIBERINFEC, Madrid, Spain

^3^ University of Vic – Central University of Catalonia (UVic – UCC), Vic, Catalonia, Spain

^4^ Universitat Autonoma de Barcelona (UAB), Barcelona, Catalonia,Spain

^5^ Center for Global Health and Diseases, Department of Pathology, Case Western Reserve University, Cleveland, OH, United States of America

^6^ Department of Obstetrics & Gynecology, University of Manitoba, Canada

^7^ Institut Mar d’Investigacions mediques (IMIM), CIBERONC, Barcelona, Catalonia, Spain

^8^ Infectious Diseases Service. Hospital Clinic - Institut d'Investigacions Biomèdiques August Pi i Sunyer (IDIBAPS)- University of Barcelona, Barcelona, Catalonia (Spain)

^9^ Fight AIDS Foundation, Infectious Diseases Department, Germans Trias i Pujol University Hospital, Barcelona, Catalonia, Spain

^10^ Department of Infectious Diseases Service, Germans Trias i Pujol University Hospital, Barcelona, Catalonia, Spain

^11^ Catalan Institution for Research and Advanced Studies (ICREA), Barcelona, Catalonia, Spain

^12^ Department of Medicine Solna, Center for Molecular Medicine, Karolinska Institute, Karolinska University Hospital, Stockholm, Sweden

*Correspondence should be addressed to Alessandra Borgognone ([aborgognone@irsicaixa.es](mailto:aborgognone@irsicaixa.es)) & Roger Paredes ([rparedes@irsicaixa.es](mailto:rparedes@irsicaixa.es)); Infectious Diseases Department & IrsiCaixa AIDS Research Institute, Hospital Universitari Germans Trias i Pujol, Ctra. de Canyet s/n, Planta 2a, 08916 Badalona, Catalonia, Spain.

**This Word file includes:**

Supplementary Methods

Supplementary Results

Supplementary Figures

**Supplementary Methods**

**Fecal sample collection, DNA extraction and library preparation.**

Study participants collected fecal samples at home in sterile collection tubes using standardized sampling procedures and stored immediately at approximately −20°C. Samples were then transferred to the laboratory and cryopreserved at −80°C until use. Total DNA for shotgun metagenomic sequencing was extracted and purified from each fecal sample using the PowerSoil DNA Extraction kit (MO Bio Laboratories, Carlsbad, CA), following the manufacturer’s instructions. Extracted DNA was fragmented using a Nextera-XT DNA Library Kit (Illumina, CA, USA) and one library of 300-bp average clone insert size was constructed for each sample.

**Sequencing and data quality assessment**

Metagenomic sequencing libraries were processed on an Illumina HiSeq platform (Illumina, CA, USA) by Macrogen Genomic Division. Delivered raw sequencing data were passed through the FastQC quality control software[1]. FASTQ sequence files were filtered using Trimmomatic[2] to remove Nextera adapters and low-quality reads (minimum base quality = Q30; minimum length = 75 bp), using a sliding window set at a minimum quality of Q20 for each 30-bp-long consecutive segments. Filtered reads were then mapped against the human genome using Bowtie2[3] to remove host DNA contamination. Reads uniquely aligned to the human reference with quality score above Q20 were discarded.

**Taxonomic classification and microbial gene richness**

Taxonomic profiles were characterized by MetaPhlAn2 software[4] (default parameters), which used clade-specific markers to classify metagenomic reads and quantify relative abundance of taxa within each fecal sample. Microbial gene richness was assessed using the integrated gene catalog of human gut microbiome (IGC)[5] and Bowtie2 to map filtered reads to IGC. Only filtered forward reads were used to estimate the number of unique genes and a minimum of 1 mapped read was set to consider the presence of a gene. The copy number of each gene was estimated by dividing the total reads mapping to a gene divided by the gene’s length. Gene relative abundance was measured as its copy number divided by the sum of the total gene copies in the sample, as previously reported [6]. Approximately 85% of total filtered sequences (763 835 224 reads across all samples) aligned against the IGC reference catalog (Additional file 3: Table S6). Based on the number of aligned sequences and rarefaction curve of new unique genes, a cut-off of 10 million mapped reads was used to compare microbial gene richness across samples.

**Microbial functional profiling**

Gene family abundance, metabolic pathway abundance and pathway coverage of each sample were determined from processed reads using HUMAnN2 (v0.11.1) with default parameters[7]. HUMAnN2 provides representative species-specific gene lists, using UniRef90[8], MetaCyc[9] and MinPath[10] databases combined with MetaPhlAn2 and ChocoPhlAn pangenome databases for taxonomic identification. The resulting gene families and pathway abundance files from all samples were joined and normalized to relative abundance. The functional analysis was focused on the output of pathway abundance, which provided comprehensive quantitative insight into the functional aspects of a microbial community.

**Statistical analysis of microbiome data**

Microbial diversity and composition from shotgun metagenomic data were determined using the R (v3.5.0)[11] packages *phyloseq*[12], *vegan*[13] and *ggplot2*[14] for data visualization. Alpha diversity was estimated by the Shannon index, a measure accounting for both species abundance and evenness. Principal coordinated analysis (PCoA) based on Bray-Curtis dissimilarity was performed to display sample grouping according to their microbial composition (abundance-based). PERMANOVA (*adonis*) test based on Bray-Curtis distances was performed to estimate statistical significance of group-wise beta diversity. Normalized microbial abundances derived by MetaPhlAn2 were fed into the LEfSe algorithm[15] to identify differentially abundant taxa between groups. A difference was considered statistically significant if LDA score >2 and *p-*value < 0.05 (Kruskal–Wallis test) after multiple test correction by FDR adjustment. To track longitudinal changes between consecutive time points, data obtained from MetaPhlAn2 were analyzed using q2-longitudinal plugin[16]. *Feature volatility* analysis was performed to assess longitudinal microbial abundance variations, comparing individual features within each group. Differences of intra-group comparison were assessed using paired Wilcoxon signed-rank test, whereas non-paired Wilcoxon signed-rank test was used for between-groups comparisons (two.sided) with Benjamini-Hochberg adjustment for multiple comparisons (FDR=5%). Benjamini–Hochberg multiple hypothesis correction for unadjusted p-values ≤ 0.05 were provided in Additional file 2: Dataset S7.

**Sample preparation and mass spectrometry analysis for metaproteomics**

A volume of 1.5 ml PBS was added to stool samples. Homogenization was performed by vortexing for 25 min at 4°C. A portion of the stool sample was then aliquoted and diluted further (dilution factor of 4) with PBS. Large particulates (undigested material, human cells) were pelleted out by low-speed centrifugation (300 x g for 5 min at 4°C). The supernatant was separated from the debris pellet and underwent further centrifugation (14,000 x g for 20 min at 4°C) to pellet out bacterial cells, which was then washed 3 times with PBS, vortexed, and resuspended in 250 μl of 4% SDS lysis buffer. The cells were then subjected to agitation and heat on a thermomixer (95°C for 10 min). Samples were then subjected to 3 cycles of probe sonication (1 min at 25% amplitude, 1 minute on ice), then 3 cycles of bead beating (0.1 mm silica beads). Bead beating cycles involved 60 sec of homogenization, centrifugation (14,000 x g for 5 min at RT) to pellet unlysed cells, transfer of resulting supernatant (cell lysate) into a separate tube, followed by an addition of 250 μl of lysis buffer to the unlysed cell pellet. Lysate protein content was quantified using the 2D Quant protein quantification kit (GE Healthcare Lifesciences). A trypsin digestion was carried out on 100 μg of protein via a FASP method. Urea (8 M), DTT (25 mM) and IAA (50 mM) were used for the denaturation, reduction, and alkylation of proteins, respectively. Samples were desalted using HPLC. The purified peptides were quantified using a quantitative fluorometric peptide assay (Pierce). Peptide samples were then dried down via vacuum centrifugation, and were resuspended in nano-LC buffer to a concentration of 0.25 μg/μl. An amount of 0.5 μg of peptide was injected into LC-MS for analysis. Mass spectrometry analysis of stool peptides was performed as described previously[17], using a nano-flow Easy 1000 in line to a Orbitrap Fusion Lumos mass spectrometer (Thermo Fischer Scientific). A reference pooled stool sample was run every 10 samples to monitor MS consistency. Bacterial peptides were annotated against the human gut integrated non-redundant gene catalog (CNGdb, db.cngb.org) using the Mascot search engine (v2.4, Matrix Science), with human peptides added to limit potential homologous identifications. Search results were analyzed using Scaffold Q+ software (v4.9.0, Proteome Software). Identifications were restricted to those that passed a ≤1% FDR at the protein level, ≤0.1% FDR at the peptide level, and had ≤2 unique peptides/protein. Bacterial proteins were binned to either the order or genus level, with proteins that could not be assigned to a taxon classified as “undistinguishable”. Differences in the relative abundance of taxa were assessed using Mann-Whitney U tests, with Benjamini-Hochberg adjustment for multiple comparisons (FDR=5%). Benjamini–Hochberg multiple hypothesis correction for p-values ≤ 0.05 were provided in Additional file 2: Dataset S7. Proteins were annotated for biological functions using KEGG gene ontology.

**Isolation of PBMCs, RNA library preparation and data analysis**

The transcriptome was evaluated using RNA sequencing (RNA-seq) of peripheral blood mononuclear cells (PBMCs). Blood samples were collected prior vaccination and processed with Lymphoprep (STEMCELL technologies) We used AllPrep DNA/RNA Mini Kit kit and the Qiacube standard protocol RNAeasy Mini- Animal tissues and cells to extract total RNA from 2M frozen PBMCs. Ribosomal RNA was removed from total RNA using RiboZero Magnetic Gold Kit and ribosomal RNA-depleted RNA from each sample was purified and fragmented. The RNASeq libraries from total RNA samples were prepared using a TruSeq™ Stranded Total RNA kit protocol (Illumina) according to manufacturer’s protocol. Each resultant library was quantified Agilent DNA 7500 Bioanalyzer assay (Agilent). The libraries were sequenced on HiSeq2000 (Illumina) in paired-end mode with a read length of 2x76bp using TruSeq SBS Kit v4 in a fraction of a sequencing v4 flow cell lane, following the manufacturer’s protocol. Image analysis, base calling and quality scoring of the run were processed using the manufacturer’s software Real Time Analysis (RTA 1.18.66.3) and followed by generation of FASTQ sequence files by CASAVA. High quality reads were then mapped to the hg19 human reference genome (GrCh38 version)[18] using STAR v2.5.3a aligner [19] with ENCODE parameters. The number of reads counts aligning to each gene was estimated using RSEM v1.3.0 [20] and outputs from each sample were combined into a count matrix for subsequent analyses. Estimate abundances were analyzed for differentially expressed genes (DEGs) using the geometric mean method and negative binomial generalized linear models integrated in the DESeq2[21] R package. To filter out low-expressed genes, features below a row-sum threshold of 5 were removed from the dataset. The input file contained 13 samples as columns and 58,450 genes as rows. After filtering steps, 13 samples with expression data from 27, 426 genes were obtained. Count data were transformed and normalized using DESeq2 regularized-logarithm transformation (rlog) to remove the dependence of variance on mean. A threshold of adjusted p-value < 0.1, after adjusting for multiple testing using Benjamini-Hochberg, and absolute values of log2 fold change > 0 were used to identify DEGs. Gene enrichment analysis was performed using enrichGO function from the ClusterProfiler package[22] based on the ‘biological process’ gene ontology (GO) category. The following parameters were set as cut-off criteria: organism, *Homo sapiens*; ontology, BP; universe, filtered set of genes from the study dataset; p-value cut-off, 0.05; P-adjust method, Bonferroni; readable, T. GO enrichment results were visualized using ggplot2 and enrichGO outputs were fed into REVIGO[23] to remove redundant GO terms and identify representative GO clusters.

**Targeted proteomic profiling of soluble factors in plasma**

Plasma samples were processed for analysis of soluble proteins. Concentrations of the protein profiles comprised in the Olink® Inflammation Panel (92 inflammation-related protein biomarkers, Olink Bioscience AB, Uppsala, Sweden)[24] were estimated using the Proximity Extension Assay (PEA). Briefly, the multiplex immunoassay was based on protein target-specific antibodies coupled to two single-strand oligonucleotides (proximity probes) that, upon binding to their respective epitopes, generated a target sequence for a quantitative real time PCR (qRT-PCR) reaction. The Ct values from qRT-PCR were normalized by the subtraction of values for extension control, as well as an inter-plate control, and the resulting data transformed into normalized protein expression (NPX) units. A correction factor (normal background noise) was used to report arbitrary units on log2 scale, allowing relative quantification of proteins[25]. In the original dataset, data were missing for B05 and B07 participants

**‘Omic’ data correlations**

Spearman's correlation coefficients were computed for associations between gut microbial signatures, normalized individual bacterial metaproteomic, human transcriptomic data and viral reservoir size. Spearman’s rho, corresponding *p* values and p-values adjusted for multiple comparisons by the Benjamini-Hochberg method were calculated using ‘rcorr’ function within R package *hmisc*. Correlation matrices were produced using the R package *corrplot* [26] and adjusted p-value ≤ 0.05 was considered as a significant correlation*.* Correlation-based network analysis was conducted and visualized using *qgraph* package implemented in R [27]. Spring layout was used to classify similar terms based on the strength of their connections. Functional enrichment analysis was performed using enrichGO function within the ClusterProfiler package[22]. Correlation analysis of metagenomic, transcriptomic and metaproteomic datasets was performed using the multiblock analysis DIABLO (block.splsda function) from mixOmics R package[28]. Variable selection was based on features characterized in previous steps. A correlation threshold of 0.8 was set to determine key interactions between the selected data. Correlation networks were imported and plotted using mixOmics predefined functions. Cytoscape (v3.8.2) was used to build networks from metagenomic, transcriptomic and metaproteomic signatures[29].

**Supplementary Results**

**Shotgun metagenomic sequencing analysis**

A median of 13 *vs* 11.4 million shotgun paired-end reads were generated for longitudinal samples belonging to controllers (n=23) and non-controllers (n=51), respectively. After trimming and filtering, 99.3% of sequencing data passed quality filters. A median of 12.9 and 11.3 non-human high-quality reads were obtained in viremic controllers and non-controllers, respectively (Additional file 3: Table S6). The relative abundance of microbial communities in each sample was obtained using Metaphlan2 [4]. Mapped reads were mostly attributed to bacteria (99.7% controllers *vs* 97.9 % non-controllers) and smaller proportions corresponded to archaea (0.2 % controllers *vs* 0.5 % non-controllers), viruses (0.2 % controllers *vs* 1.6 % non-controllers) and eukaryotes (0.0008 % controllers *vs* 0 % non-controllers). Consistent with previous studies [30], *Bacteroidales* (70% in controllers and 45% in non-controllers) and *Clostridiales* (21% in controllers and 33% in non-controllers) were the dominant orders in both groups, although at different proportions (Fig. S3). *Erysipelotrichales*, *Selenomonadales*, *Lactobacillales* and *Bifidobacteriales* were detected longitudinally at lower proportions (5,3%, 4,3%, 3,2% and 2,1% in non-controllers *vs* 3.1%, 1,3%, 0,5% and 0,5% in controllers) (Fig. S3). Relative abundances per each time point are outlined in Additional file 2: Dataset S1.

**No significant alteration in microbial composition and diversity during MAP and after ART re-initiation**

All 9 non-controllers analyzed in this study resumed ART by week 4 after the MAP initiation, whereas the 3 controllers remained off ART for at least 28 weeks and up to 32 weeks. In the assessment of bacterial order abundance, Bacteroidales showed an initial increase up to week 4 followed by a reduction by weeks 8-12 in controllers (Fig. 1a) during the MAP. Inversely, Clostridiales levels increased by weeks 8-12 and remained stable thereafter (Fig. 1b) (no statistical testing provided during MAP). No significant differences in bacterial composition were found following ART resumption; however, there was a limited sample availability at ART resumption phase (Figs. 1a-c). Longitudinal evaluation also showed that gut microbial diversity increased around weeks 8-10 in controllers, and remained stable thereafter (Figs. 2a-b) during the MAP. Whereas, no statistically significant differences were found in microbial diversity following ART re-initiation (Figs. 2a-b).

**Longitudinal microbial metabolic pathways differ between viremic controllers and non-controllers**

Functional profiling based on HUMAnN2 identified 22 differential metabolic pathways related to *Bacteroidales* and *Clostridiales* between controllers and non-controllers at study entry (unadjusted *p-*valu*e* <0.05) (Figs. S9 and S10a). Thirteen out of the 22 pathways identified at study entry were differentially abundant over the intervention (Fig. S10b). Controllers were mainly enriched in pathways related to fatty acid, lipid and amino acid biosynthesis. Conversely, metabolic pathways related to carbohydrate biosynthesis were overrepresented in non-controllers. Longitudinal variations of such metabolic pathways during MAP and after ART re-initiation are shown (Fig. S11), although low numbers did not allow for statistical testing. Moreover, functions related to methanogenesis from the archaeal species *Methanobrevibacter smithii* and *Methanosphaera stadtmanae* largely discriminated between controllers and non-controllers (fold-change=11.5, *p*=0.046 at study entry, Fig. S12a), showing significant enrichment in non-controllers throughout the intervention (Fig. S12b). Consistently with profiles in Fig. S4, both methanogenic *Methanobrevibacter smithii* and *Methanosphaera stadtmanae* species were detected in most non-controllers but were rare or absent in controllers over the intervention (Fig. S12c).

**Increased baseline inflammation-related plasma proteins in viremic controllers**

Soluble factors in plasma from the 92 inflammation-related protein panel were assessed using the Proximity Extension Assay. Plasma protein levels did not independently separate controllers from non-controllers using unbiased hierarchical clustering or principal component analysis (Figs. S15a-b). Of the 92 plasma proteins characterized (Additional file 2: Dataset S4), only 7 were differentially expressed (Wilcoxon, uncorrected *p*<0.05) and were increased in controllers (Fig. S15c): adenosine deaminase ADA (*p*=0.012), decoy receptor osteoprotegerin OPG (*p*=0.024), self-ligand receptor of the signaling lymphocytic activation molecule family SLAMF-1 (*p*=0.048), chemokines CCL23, CCL28, MCP-2 (*p*=0.048) and the neurotrophic factor NT-3 (*p*=0.048) (Fig. S15d).

**Host transcript correlated with *Bacteroidales/Clostridiales* ratio and viral reservoir showed functional enrichment in immune activation**

Enrichment analysis extended the complete set of the host transcripts significantly correlated with the ratio *Bacteroidales/Clostridiales* (n. transcripts=453, *p_adj_* <0.05, Additional file 2: Dataset S5) showed functional enrichment in immune system activation and host defense (Fig. S16a and Additional file 3: Table S4). Moreover, strong correlations were observed between the *Bacteroidales/Clostridiales* ratio and transcripts involved in the enrichment analysis (rho >0.8 in Additional file 2: Dataset S5), mostly highly expressed in controllers (Fig. 6b). Of the 453 transcripts significantly correlated with the *Bacteroidales/Clostridiales* ratio, 61 and 70 also correlated with the baseline CA HIV-1 RNA and HIV-1 DNA, respectively (rho = 0.5; Additional file 2: Dataset S6), and were enriched in immune-mediated response functions (Figs. S16b-c and Additional file 3: Table S5).

**References**

1. Andrews S, Krueger F, Seconds-Pichon A, Biggins F, Wingett S. FastQC. A quality control tool for high throughput sequence data. Babraham Bioinformatics [Internet]. Babraham Inst. 2015. p. 1. Available from: https://www.bioinformatics.babraham.ac.uk/projects/fastqc/%0Ahttp://www.bioinformatics.bbsrc.ac.uk/projects/fastqc/

2. Bolger AM, Lohse M, Usadel B. Trimmomatic: a flexible trimmer for Illumina sequence data. Bioinformatics [Internet]. Oxford University Press; 2014 [cited 2017 Feb 22];30:2114–20. Available from: https://academic.oup.com/bioinformatics/article-lookup/doi/10.1093/bioinformatics/btu170

3. Langmead B, Salzberg SL. Fast gapped-read alignment with Bowtie 2. Nat Methods. 2012;9:357–9.

4. Truong DT, Franzosa EA, Tickle TL, Scholz M, Weingart G, Pasolli E, et al. MetaPhlAn2 for enhanced metagenomic taxonomic profiling. Nat. Methods. 2015. p. 902–3.

5. Li J, Wang J, Jia H, Cai X, Zhong H, Feng Q, et al. An integrated catalog of reference genes in the human gut microbiome. Nat Biotechnol. 2014;32:834–41.

6. Le Chatelier E, Nielsen T, Qin J, Prifti E, Hildebrand F, Falony G, et al. Richness of human gut microbiome correlates with metabolic markers. Nature. 2013;500:541–6.

7. Franzosa EA, McIver LJ, Rahnavard G, Thompson LR, Schirmer M, Weingart G, et al. Species-level functional profiling of metagenomes and metatranscriptomes. Nat Methods. 2018;15:962–8.

8. Suzek BE, Wang Y, Huang H, McGarvey PB, Wu CH. UniRef clusters: A comprehensive and scalable alternative for improving sequence similarity searches. Bioinformatics. 2015;31:926–32.

9. Caspi R, Billington R, Fulcher CA, Keseler IM, Kothari A, Krummenacker M, et al. The MetaCyc database of metabolic pathways and enzymes. Nucleic Acids Res. 2018;46:D633–9.

10. Ye Y, Doak TG. A parsimony approach to biological pathway reconstruction/inference for genomes and metagenomes. PLoS Comput Biol. 2009;5.

11. R Foundation for Statistical Computing. R: a Language and Environment for Statistical Computing. http://www.R-project.org/. 2018.

12. McMurdie PJ, Holmes S. Phyloseq: An R Package for Reproducible Interactive Analysis and Graphics of Microbiome Census Data. PLoS One. 2013;8.

13. Oksanen J, Blanchet FG, Friendly M, Kindt R, Legendre P, McGlinn D, et al. vegan: Community Ecology Package. R package version 2.5-2. Cran R [Internet]. 2019;1:2. Available from: https://cran.r-project.org/package=vegan

14. Wickham H. Package `ggplot2`: Elegant Graphics for Data Analysis. Springer-Verlag New York [Internet]. 2016;1–222. Available from: https://cran.r-project.org/web/packages/ggplot2/ggplot2.pdf

15. Segata N, Izard J, Waldron L, Gevers D, Miropolsky L, Garrett WS, et al. Metagenomic biomarker discovery and explanation. Genome Biol. 2011;12.

16. Bokulich NA, Dillon MR, Zhang Y, Rideout JR, Bolyen E, Li H, et al. q2-longitudinal: Longitudinal and Paired-Sample Analyses of Microbiome Data. mSystems. 2018;3.

17. Klatt NR, Cheu R, Birse K, Zevin AS, Perner M, Noël-Romas L, et al. Vaginal bacteria modify HIV tenofovir microbicide efficacy in African women. Science (80- ). 2017;356:938–45.

18. Pruitt KD, Tatusova T, Maglott DR. NCBI Reference Sequence (RefSeq): A curated non-redundant sequence database of genomes, transcripts and proteins. Nucleic Acids Res. 2005;33.

19. Dobin A, Davis CA, Schlesinger F, Drenkow J, Zaleski C, Jha S, et al. STAR: ultrafast universal RNA-seq aligner. Bioinformatics [Internet]. 2013 [cited 2017 Feb 22];29:15–21. Available from: http://www.ncbi.nlm.nih.gov/pubmed/23104886

20. Li B, Dewey CN. RSEM: Accurate transcript quantification from RNA-Seq data with or without a reference genome. BMC Bioinformatics. 2011;12.

21. Love MI, Huber W, Anders S. Moderated estimation of fold change and dispersion for RNA-seq data with DESeq2. Genome Biol. 2014;15.

22. Yu G, Wang LG, Han Y, He QY. ClusterProfiler: An R package for comparing biological themes among gene clusters. Omi A J Integr Biol. 2012;16:284–7.

23. Supek F, Bošnjak M, Škunca N, Šmuc T. Revigo summarizes and visualizes long lists of gene ontology terms. PLoS One. 2011;6.

24. Assarsson E, Lundberg M, Holmquist G, Björkesten J, Thorsen SB, Ekman D, et al. Homogenous 96-plex PEA immunoassay exhibiting high sensitivity, specificity, and excellent scalability. PLoS One. 2014;9.

25. Berggrund M, Ekman D, Gustavsson I, Sundfeldt K, Olovsson M, Enroth S, et al. Protein Detection Using the Multiplexed Proximity Extension Assay (PEA) from Plasma and Vaginal Fluid Applied to the Indicating FTA Elute Micro Card^TM^. J Circ Biomarkers. 2016;5.

26. Wei T. corrplot: Visualization of a correlation matrix. R package version 0.73. URL https//github com/taiyun/corrplot [Internet]. 2013; Available from: https://github.com/taiyun/corrplot

27. Epskamp S, Cramer AOJ, Waldorp LJ, Schmittmann VD, Borsboom D. Qgraph: Network visualizations of relationships in psychometric data. J Stat Softw. 2012;48.

28. Rohart F, Gautier B, Singh A, Lê Cao KA. mixOmics: An R package for ‘omics feature selection and multiple data integration. PLoS Comput Biol. 2017;13.

29. Shannon P, Markiel A, Ozier O, Baliga N, Wang J, Ramage D, et al. Cytoscape: A Software Environment for Integrated Models. Genome Res [Internet]. 2003;13:2498–504. Available from: http://ci.nii.ac.jp/naid/110001910481/

30. Arumugam M, Raes J, Pelletier E, Le Paslier D, Yamada T, Mende DR, et al. Enterotypes of the human gut microbiome. Nature. 2011;473:174–80.

**Supplementary Figures**

**
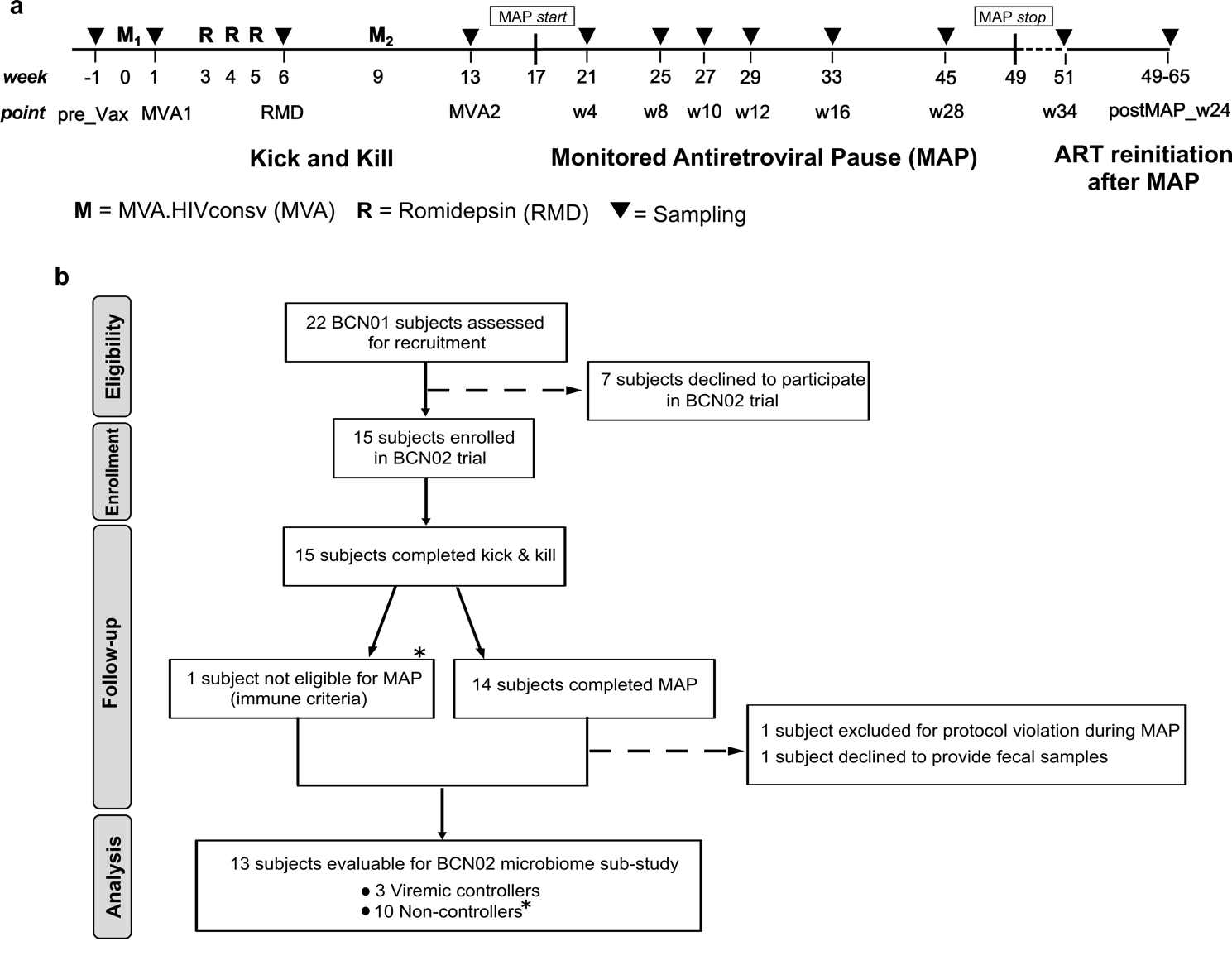
**

**Figure S1**. **BCN02-microbiome study design and sample collection strategy. a**, BCN02 trial participants were immunized with two doses of MVA.HIVconsv vaccine, before (M_1_) and after (M_2_) three weekly-doses of romidepsin (RMD) followed by a monitored antiretroviral pause (MAP) for a period of 32 weeks (or until any ART resumption criteria were met) to assess the ability to contain viral rebound after ART interruption. Samples for the BCN02-microbiome sub-study were collected at baseline (pre-Vax), during the kick and kill intervention (after M_1_, RMD_1-2-3_ and M_2_), over MAP (from 4 to 34 weeks after ART interruption) and 24 weeks after ART resumption. Timepoints included in this sub-study are indicated by black filled triangles. **b**, 13 out of the 15 BCN02 participants were rolled over the BCN02-microbiome sub-study. * One participant (B07) not eligible for MAP due to pre-defined immune futility criteria in BCN02 trial (criteria for MAP exclusion included pVL over 2,000 copies/ml in two consecutive determinations, CD4+cell counts decrease over 50% and/or below 500 cells/mm3 and/or development of clinical symptoms suggestive of an acute retroviral syndrome) was considered a non-controller in this microbiome sub-study. Abbreviations: ART, antiretroviral therapy; MAP, monitored antiretroviral pause; pVL, plasma HIV-1 viral load; 6m, 6 months; 3yr, 3 years.


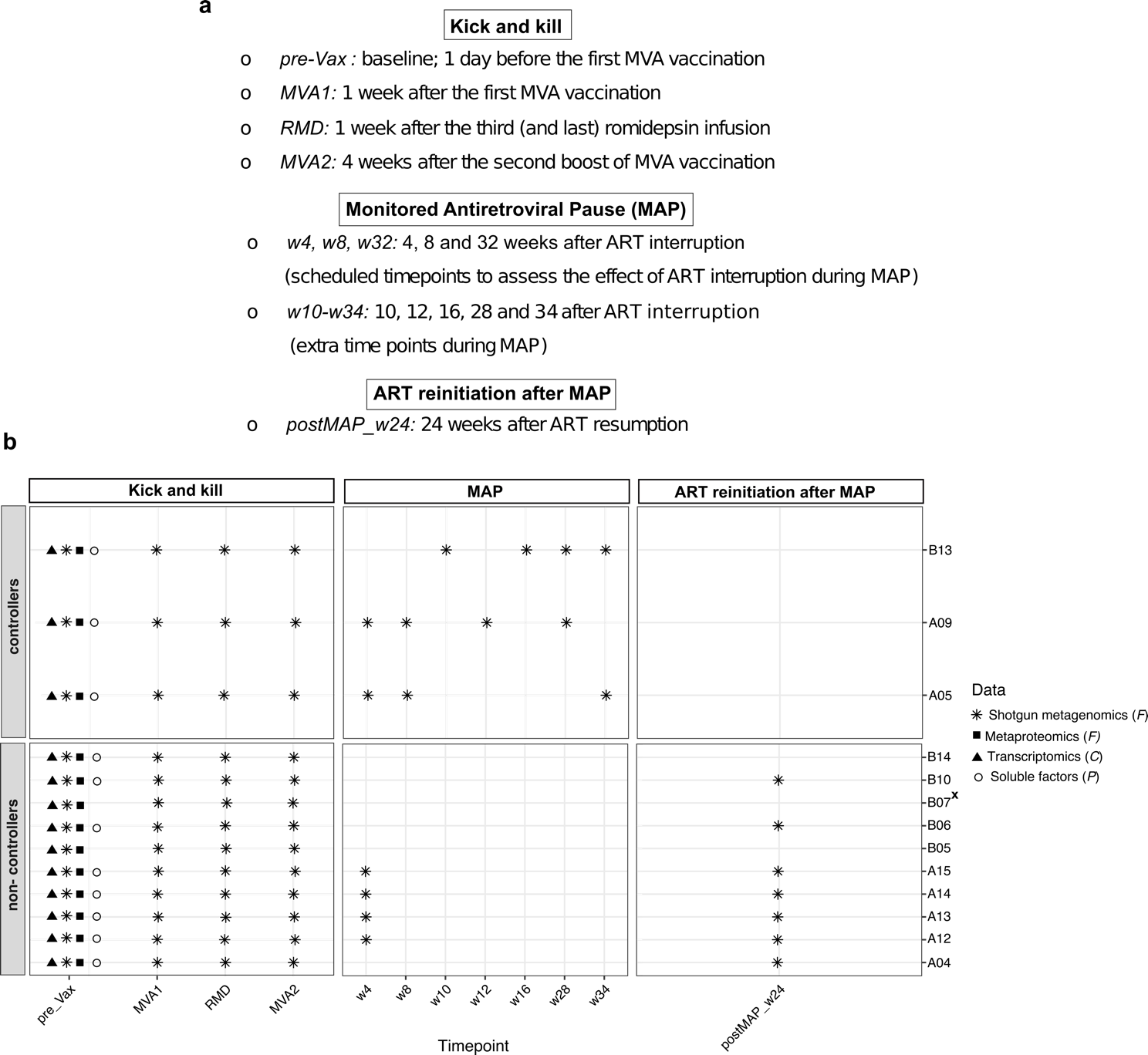


**Figure S2. Overview of sample disposition for multi-omic analysis.** **a**, Schematic representation of sampling in the BCN02-microbiome sub-study. **b**, Longitudinal sampling for multi-omics profiling including shotgun metagenomics, metaproteomics, transcriptomics and plasma proteins. In the right y-axis, participant internal identifiers are showed. Omic data categories are indicated in the legend along with the type of biological material they were performed on (F=feces, C=Peripheral blood mononuclear cells and P=plasma). (x) This participant did not enter the MAP period due to immune futility pre-defined criteria and absence beneficial HLA allele associated with natural HIV control.


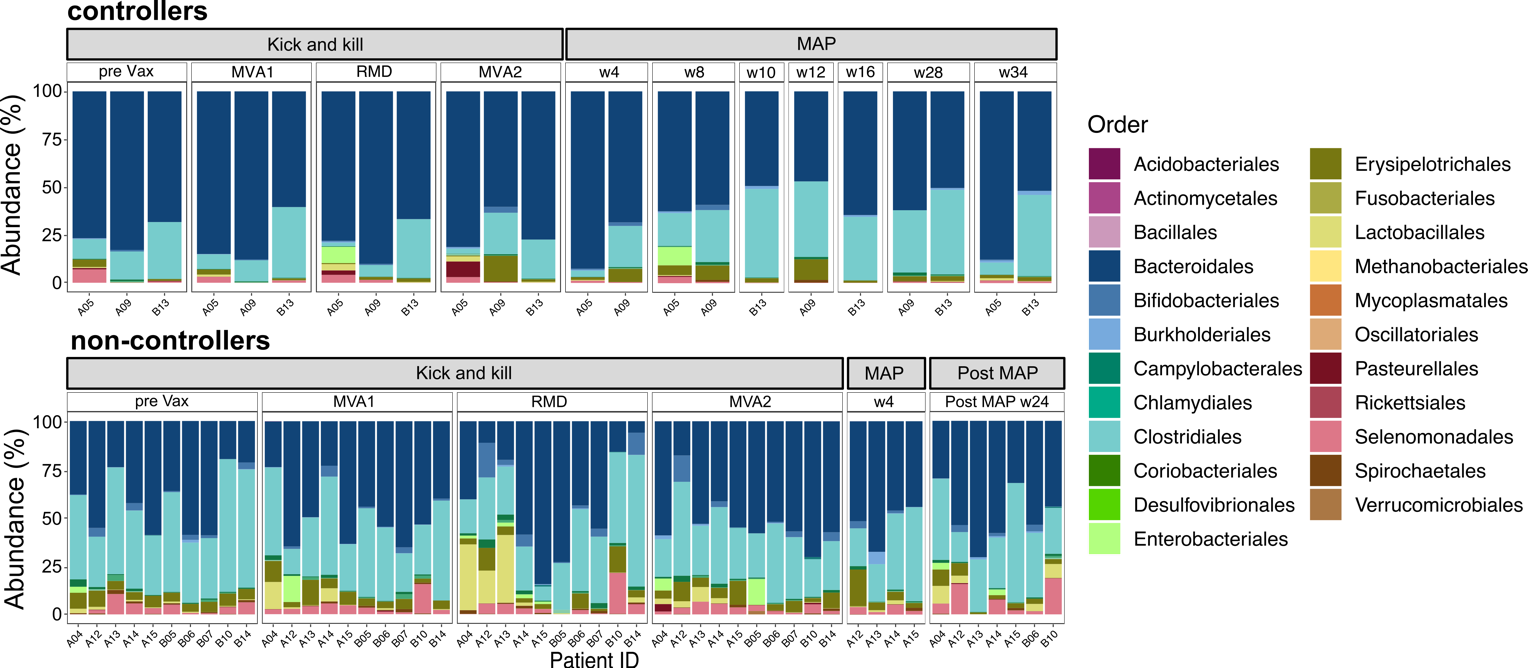


**Figure S3. Taxonomic classification of fecal samples at the order level.** Stacked bar charts showing relative abundances (in percentage) of bacterial orders at different time points for each sample within controllers and non-controllers. Each vertical bar corresponds to a study participant. Participant internal identifiers are indicated in the x-axis. Time points and phases of the trial are indicated on the top panel of each bar graph. Post MAP data are referred only to non-controllers arm. *Bacteroidales* were most represented among controllers, whereas *Clostridiales* were dominated the microbial composition in non-controllers. Abbreviations: MAP, monitored antiretroviral pause; pre_Vax, baseline (1 day before first MVA vaccination); MVA1, 1 week after first MVA vaccination; RMD, 1 week after third romidepsin infusion; MVA2, 4 weeks after second MVA vaccination.


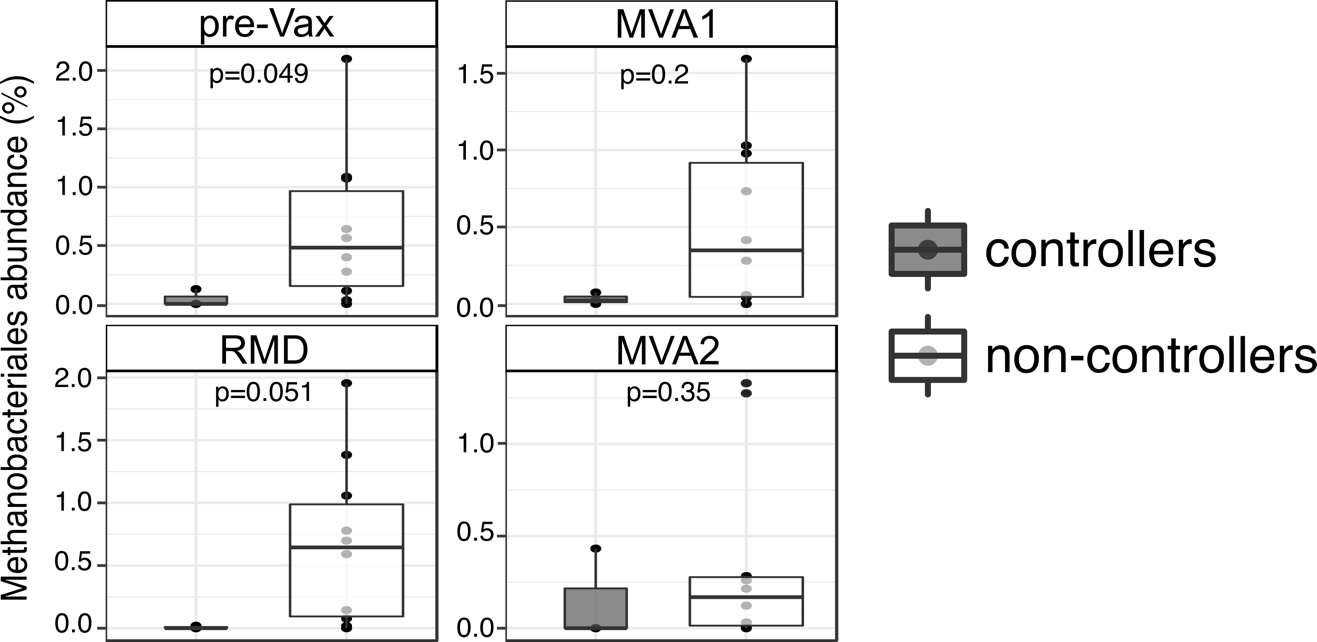


**Figure S4. Differential abundance in *Methanobacteriales* between controllers and non-controllers**. Boxplots displaying differentially abundant archaeal orders at baseline (pre-Vax) and over the ‘kick and kill’ intervention (MVA1, RMD, MVA2). Boxes indicate the interquartile range (IQR) between the first (25^th^) and third (75^th^) quartile with the median as a vertical line inside each box. Samples and outliers are displayed with dots. Abbreviations: pre-Vax, baseline (1 day before first MVA vaccination); MVA1, 1 week after first MVA vaccination; RMD, 1 week after third romidepsin infusion; MVA2, 4 weeks after second MVA vaccination. Unadjusted p-values are shown. Benjamini–Hochberg multiple hypothesis correction for *p*-values ≤ 0.05 are provided in Additional file 2: Dataset S7.


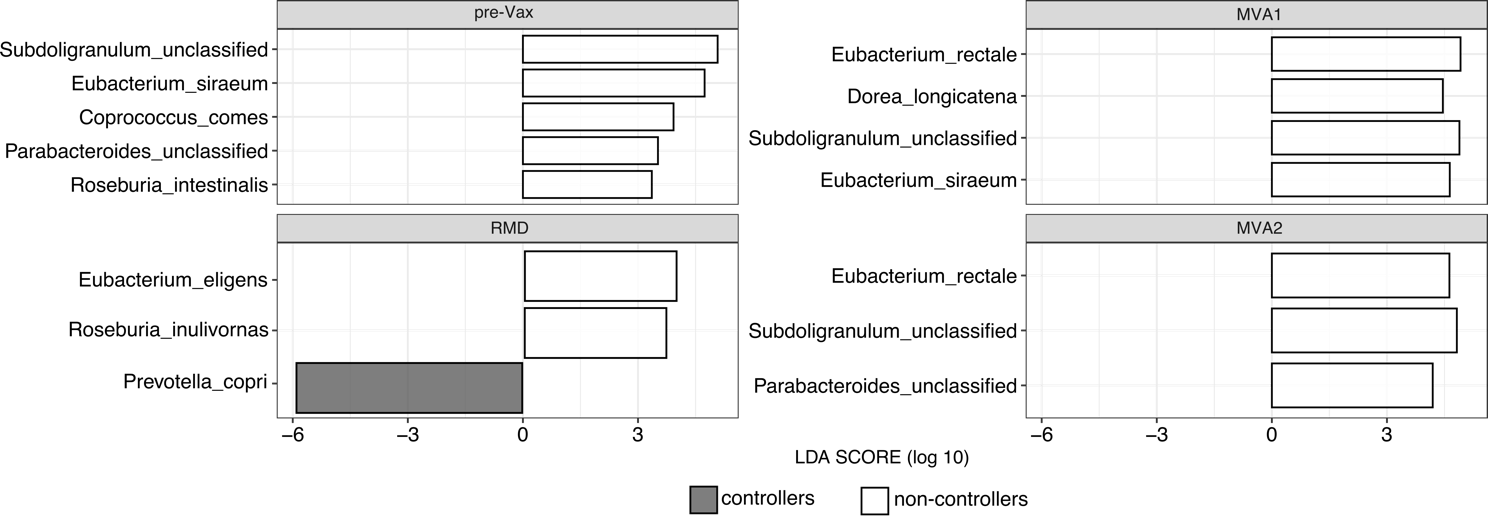


**Figure S5. Linear Discriminant Analysis (LDA) effect size (LEfSe) at the species level.** Discriminant *Bacteroidales* and *Clostridiales* species between controllers and non-controllers at baseline (pre-Vax) and during the ‘kick and kill’ intervention’**.** Enrichments in controllers and non-controllers are shown as grey bars with negative and white bars with positive LDA score, respectively. LDA score indicates the effect size and ranking of each discriminant feature. A p-value of < 0.05 and LDA score > 2 were considered significant in Kruskal–Wallis and pairwise Wilcoxon tests. Abbreviations: pre-Vax, baseline (1 day before first MVA vaccination); MVA1, 1 week after first MVA vaccination; RMD, 1 week after third romidepsin infusion; MVA2, 4 weeks after second MVA vaccination.

**~~
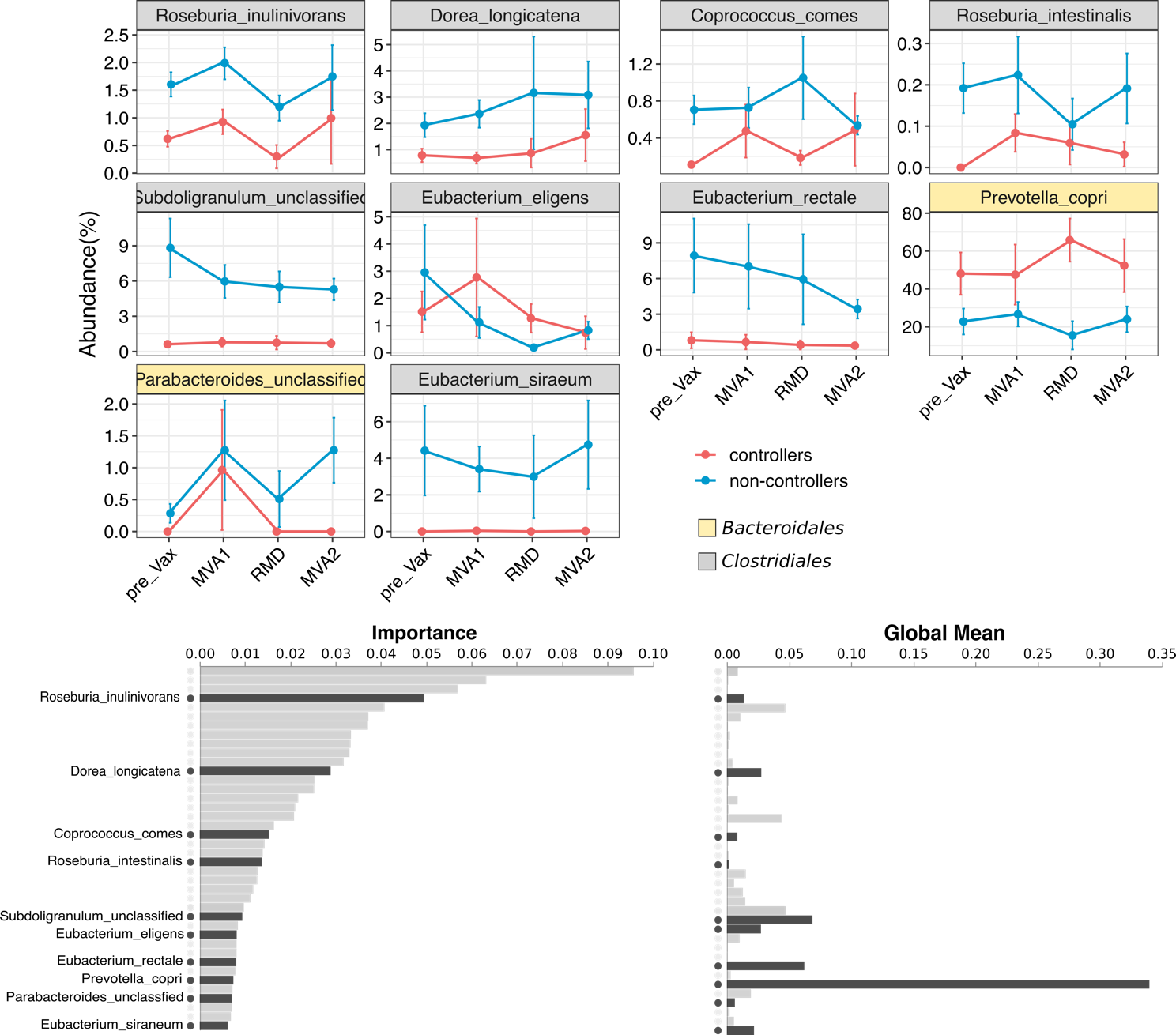
~~**

**Figure S6. Longitudinal feature-volatility analysis of *Bacteroidales* and *Clostridiales* species**. Feature-volatility results from q2-longitudinal analysis are plotted as volatility charts to visualize percentage relative abundance and bar charts to visualize features importance and global means. Global values from species identified by LEfSe analysis are represented for controllers (red) and non-controllers (blue) at baseline (pre-Vax) and over the ‘kick and kill’ intervention (MVA2). Solid lines represent the global group mean with standard deviations (± sd) from the mean at each time point. Dark gray horizontal bars indicate ‘Importance’ and ‘Global mean’ values for selected bacterial species. Abbreviations: pre-Vax, baseline (1 day before first MVA vaccination); MVA1, 1 week after first MVA vaccination; RMD, 1 week after third romidepsin infusion; MVA2, 4 weeks after second MVA vaccination.


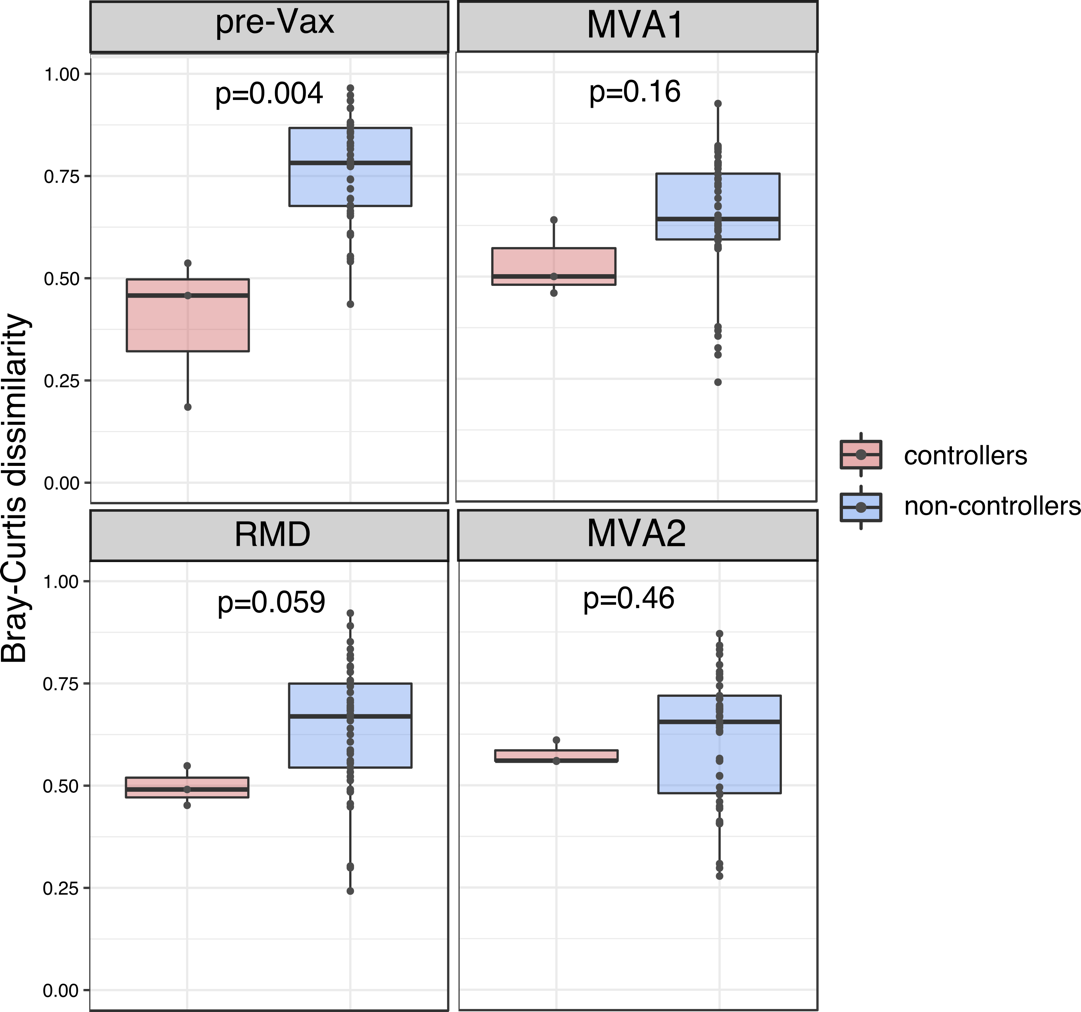


**Figure S7. Bray-Curtis dissimilarity index between controllers and non-controllers.** Comparison of Bray-Curtis dissimilarities at baseline (pre-Vax) and over the ‘kick and kill’ intervention revealed lower community dissimilarity in controllers. Boxes represent the interquartile range (IQR), the black line inside the box defines the median and whiskers represent the lowest and highest values within 1.5 IQR. Significance levels are indicated in each panel. Abbreviations: pre_Vax, baseline (1 day before first MVA vaccination); MVA1, 1 week after first MVA vaccination; RMD, 1 week after third romidepsin infusion; MVA2, 4 weeks after second MVA vaccination. Unadjusted p-values are shown. Benjamini–Hochberg multiple hypothesis correction for *p*-values ≤ 0.05 are provided in Additional file 2: Dataset S7.


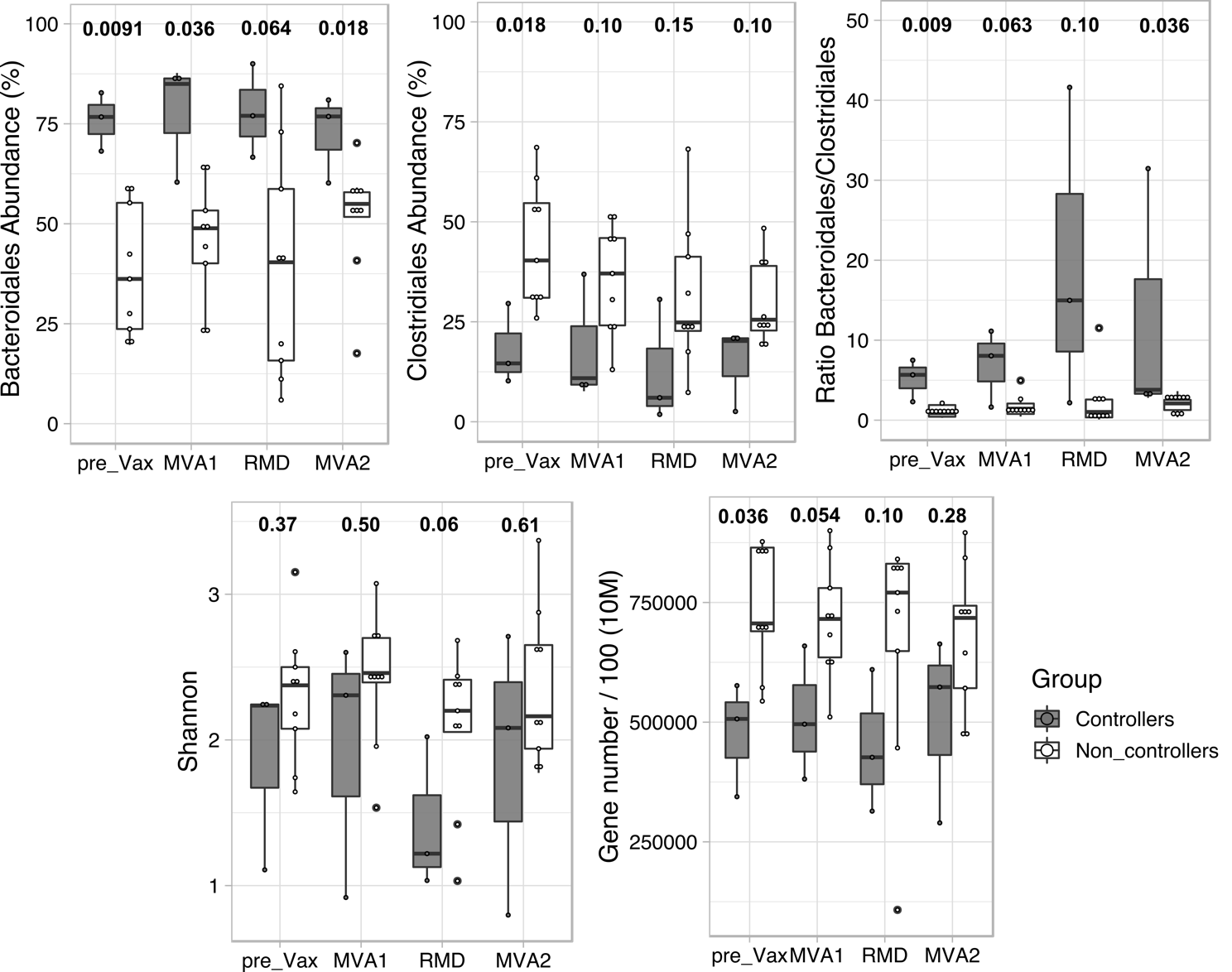


**Figure S8. Gut microbiome profiling excluding B07 participant from non-controllers arm.** Boxplots displaying longitudinal comparison (study entry and kick and kill intervention) of bacterial relative abundance (*Bacteroidales*, *Clostridiales* and their ratio), alpha diversity (Shannon index) and gene richness (downsampling at 10 Million reads) between controllers and non-controllers. B07 participant did not enter the MAP period due to immune futility pre-defined criteria and absence protective HLA allele associated with natural HIV control.

**
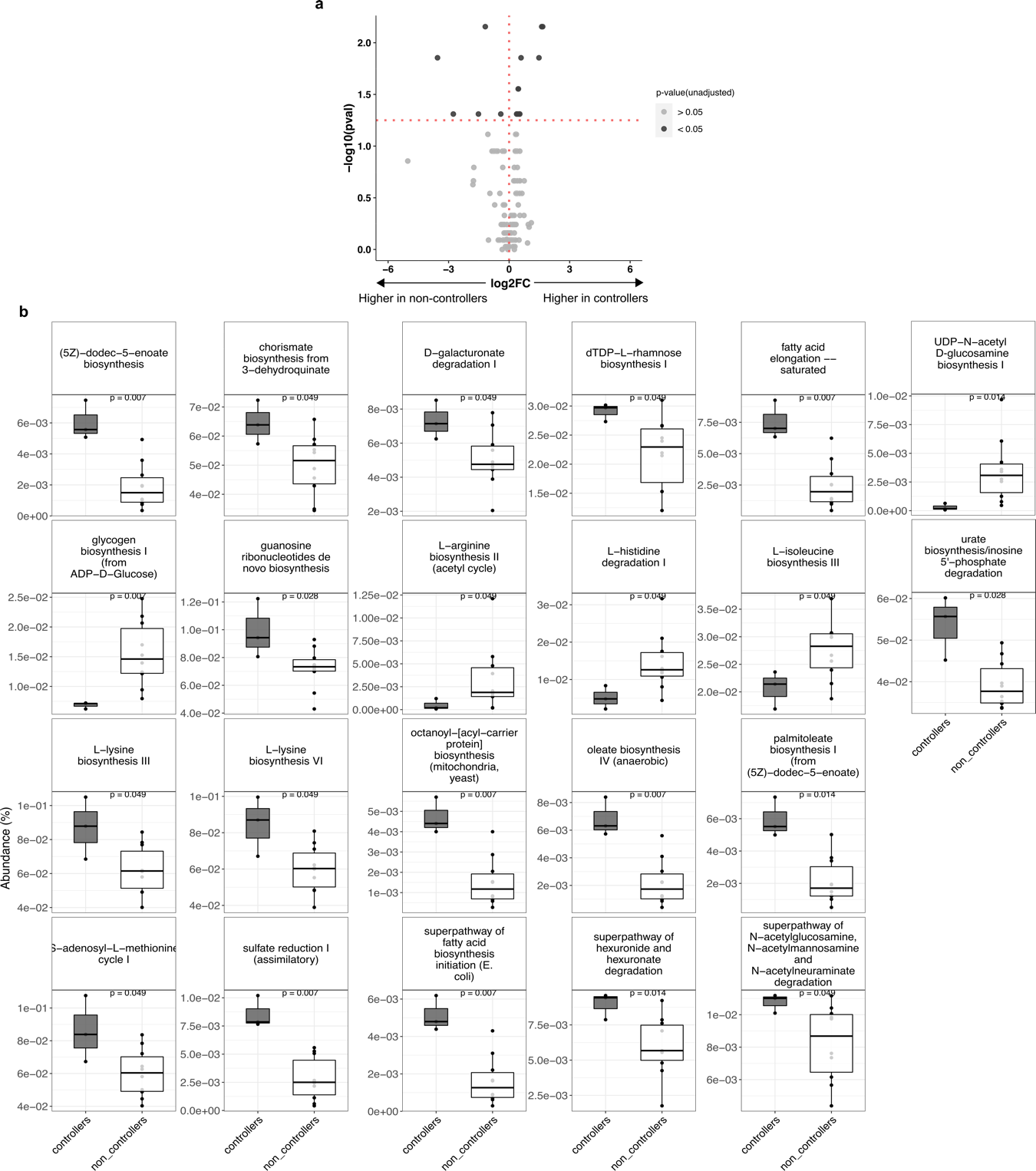
**

**Figure S9. Differentially abundant metabolic pathways from *Bacteroidales* and *Clostridiales* at the study entry. a**, Volcano plot of metabolic pathways associated to *Bacteroidales* and *Clostridiales* detected in the gut microbiome of controllers and non-controllers. Log2Fold change of pathway abundance are plotted against –log10 p-value. Dotted lines in the x-axis denotes a zero-fold change, while dotted lines in the y-axis delimit a p-value value of 0.05. **b**, Boxplots showing differential metabolic pathways between controllers and non-controllers at the BCN02 study entry (unadjusted *p* ≤ 0.05; Wilcoxon rank-sum test). Boxes represent the interquartile range (IQR) between the first and third quartiles (25^th^ and 75^th^ percentiles, respectively) and the black line inside each box indicate the median. Unadjusted p-values are shown. Benjamini–Hochberg multiple hypothesis correction for *p*-values ≤ 0.05 are provided in Additional file 2: Dataset S7.


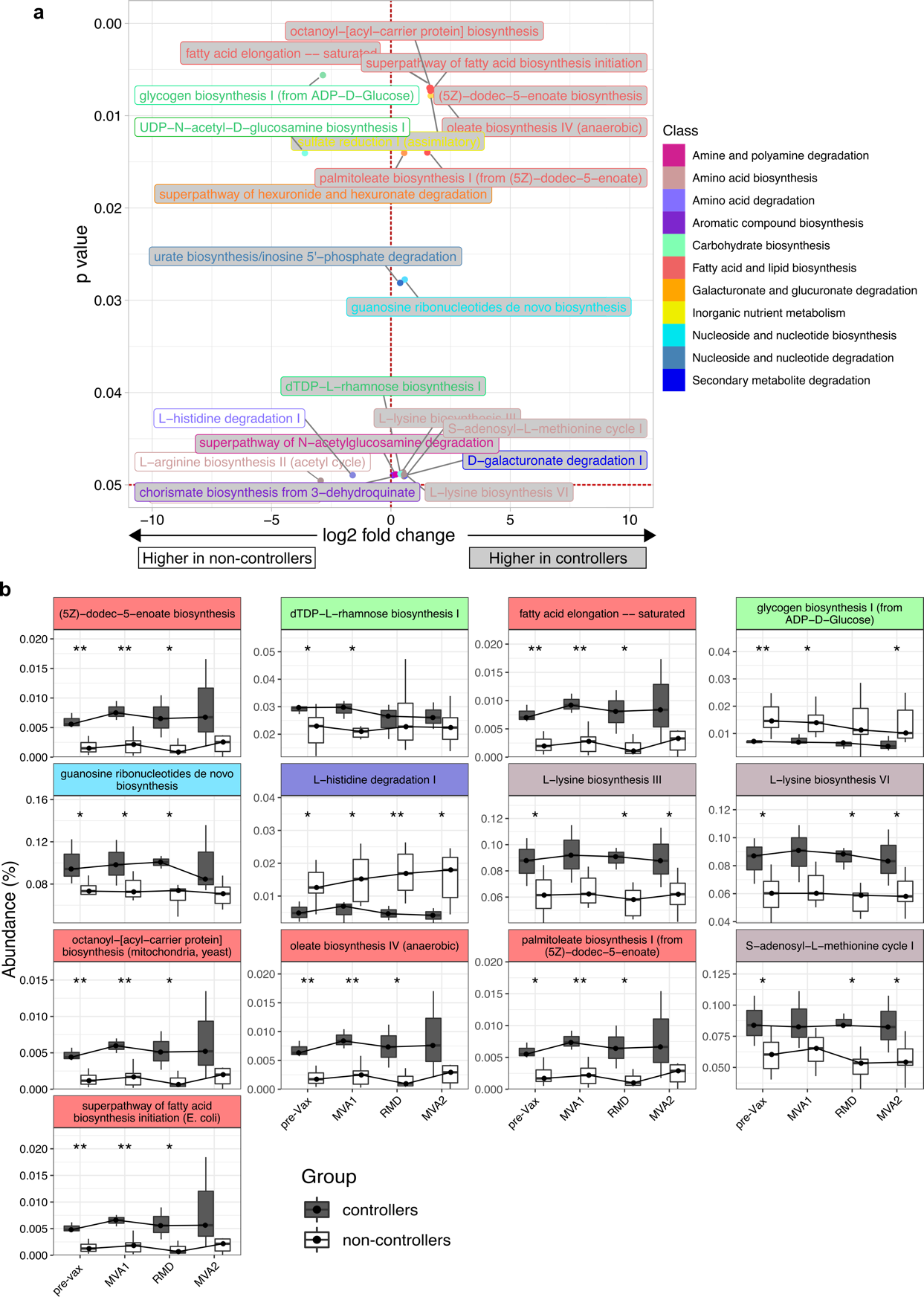


**Figure S10. Differential metabolic pathways between controllers and non-controllers. a**, Plot showing the magnitude of change of differentially abundant metabolic pathways from *Bacteroidales* and *Clostridiales* identified at pre-Vax by HUMAnN2 and grouped by classes. Log2-transformed fold change (FC) relative abundance is plotted on the x-axis and p-values are reported on the y-axis. The red horizontal line represents the p-value cutoff at 0.05. The red vertical line delineates pathways overrepresented in non-controllers (positive FC values) and controllers (negative FC values). Functions are color-coded according to classes defined by MetaCyc. **b**, Longitudinal variation of functional pathways displaying significative differences at pre-Vax and at least one additional time point over the intervention. Black solid lines link the median value of sequential time points. Pathways are color-coded by each corresponding class. Black asterisks indicate significance (unadjusted *p*-value: **p* < 0.05; ***p* < 0.01; ****p* < 0.001). Abbreviations: pre-Vax, baseline (1 day before first MVA vaccination); MVA1, 1 week after first MVA vaccination; RMD, 1 week after third romidepsin infusion; MVA2, 4 weeks after second MVA vaccination. Unadjusted p-values are shown. Benjamini–Hochberg multiple hypothesis correction for *p*-values ≤ 0.05 are provided in Additional file 2: Dataset S7.


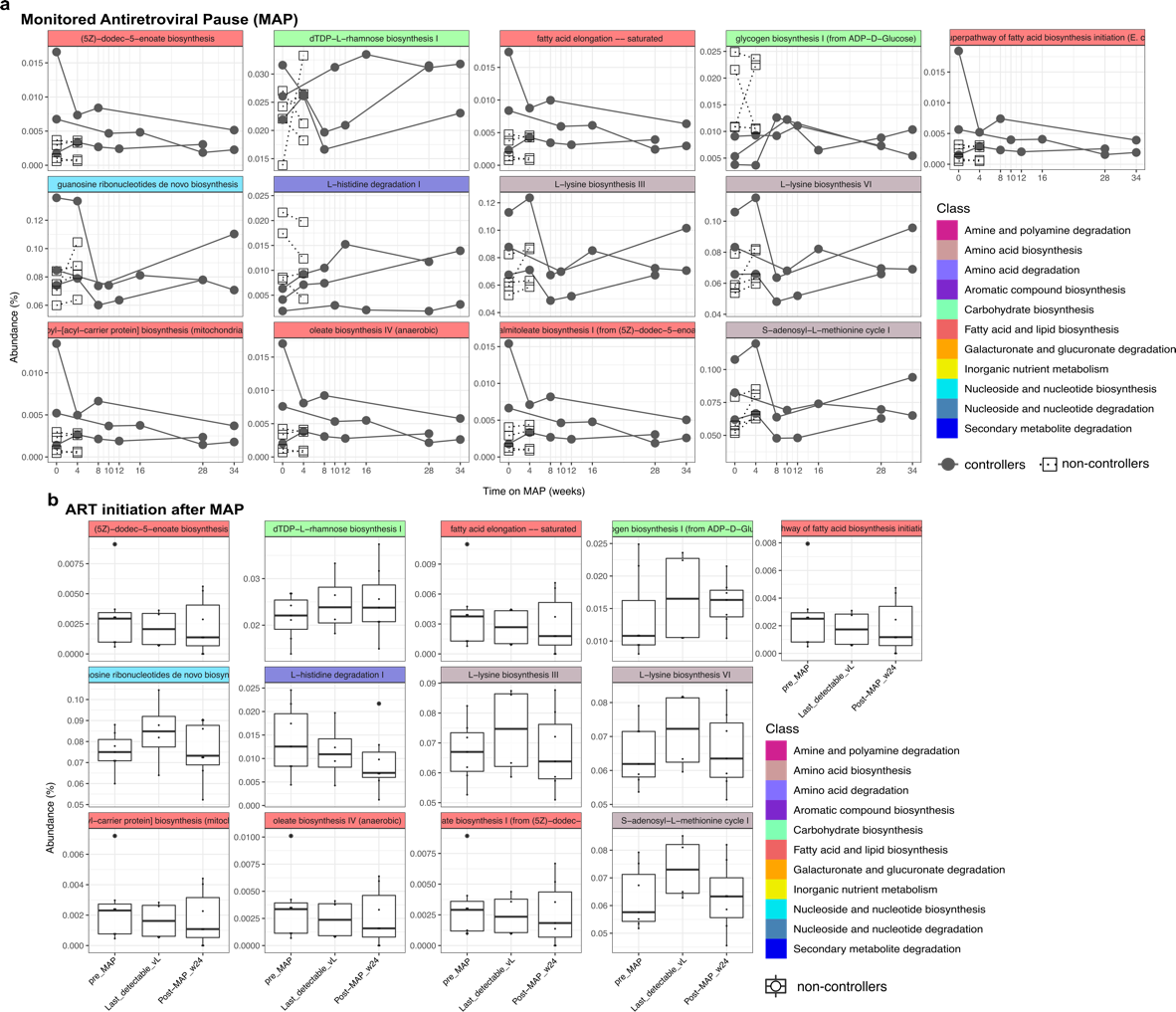


**Figure S11. Longitudinal variation of differentially abundant pathways over the trial.** Metabolic pathways from *Bacteroidales* and *Clostridiales* showing significative differences at pre-Vax and at least one additional time point during kick and kill intervention were evaluated **a**, during ART interruption (MAP) and **b**, after ART reinitiation. Line plots depict pathway relative abundances for each participant belonging to controllers (grey dots) and non-controllers (white squares) arms. Boxplots illustrate pathways relative abundances in non-controllers. Each box shows the median (horizontal black line) and interquartile range between the first and third quartiles (25^th^ and 75^th^, respectively). Pathways are color-coded by each corresponding class.


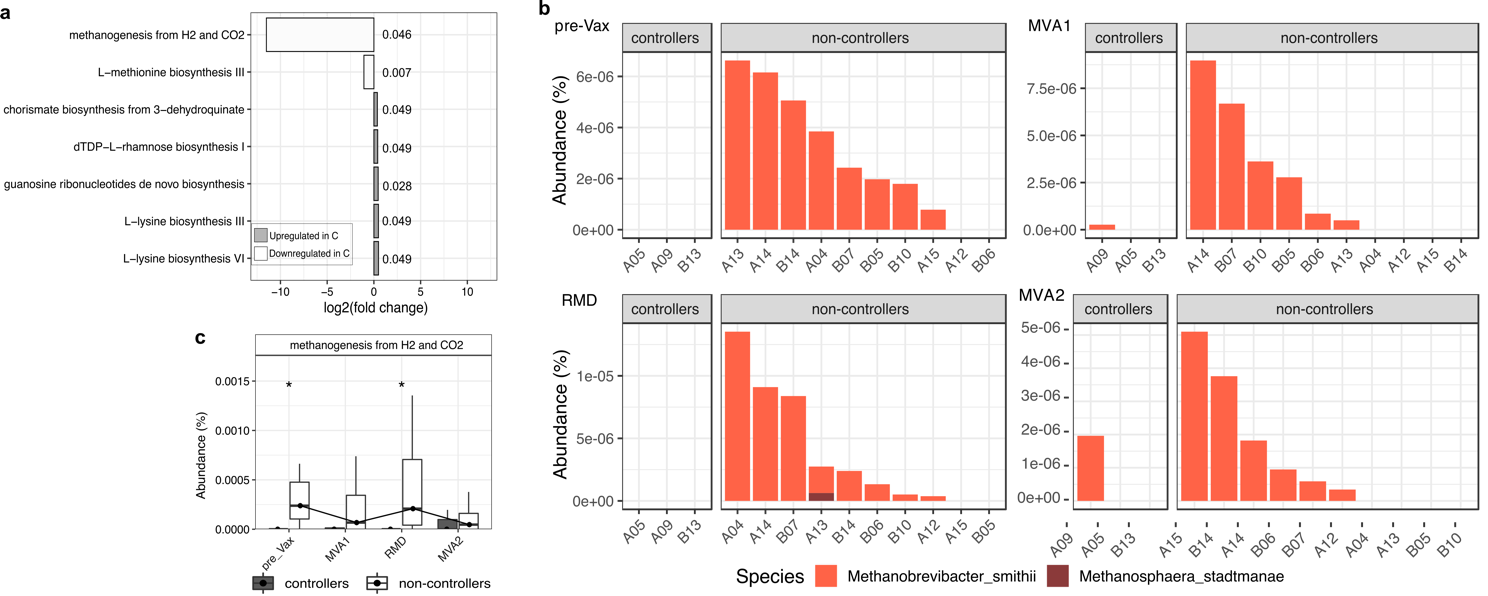


**Figure S12. Differential archaeal metabolic pathways between controllers and non-controllers. a**, Differentially abundant archaeal metabolic pathways identified at study entry and sorted by log2 (fold change). Unadjusted *p*-values are indicated next to each bar. **b**, Longitudinal variations of the ‘methanogenesis from H_2_ and CO_2_’ pathway over the intervention. Black asterisks indicate significance (unadjusted *p*-value: **p* < 0.05). **c**, Longitudinal contribution of archaeal species to the ‘methanogenesis from H_2_ and CO_2_’ pathway. Abbreviations: pre-Vax, baseline (1 day before first MVA vaccination); MVA1, 1 week after first MVA vaccination; RMD, 1 week after third romidepsin infusion; MVA2, 4 weeks after second MVA vaccination. Unadjusted p-values are shown. Benjamini–Hochberg multiple hypothesis correction for *p*-values ≤ 0.05 are provided in Additional file 2: Dataset S7.

**Figure S13. Spearman’s correlation between clinical data, vaccine response and gut microbial variables.** Positive correlations are indicated in blue and negative correlations, in red. Color and size of the circles indicate the magnitude of the correlation. White asterisks indicate significant correlations (**p* < 0.05; ***p* < 0.01; ****p* < 0.001, Benjamini–Hochberg adjustment for multiple comparisons).

**
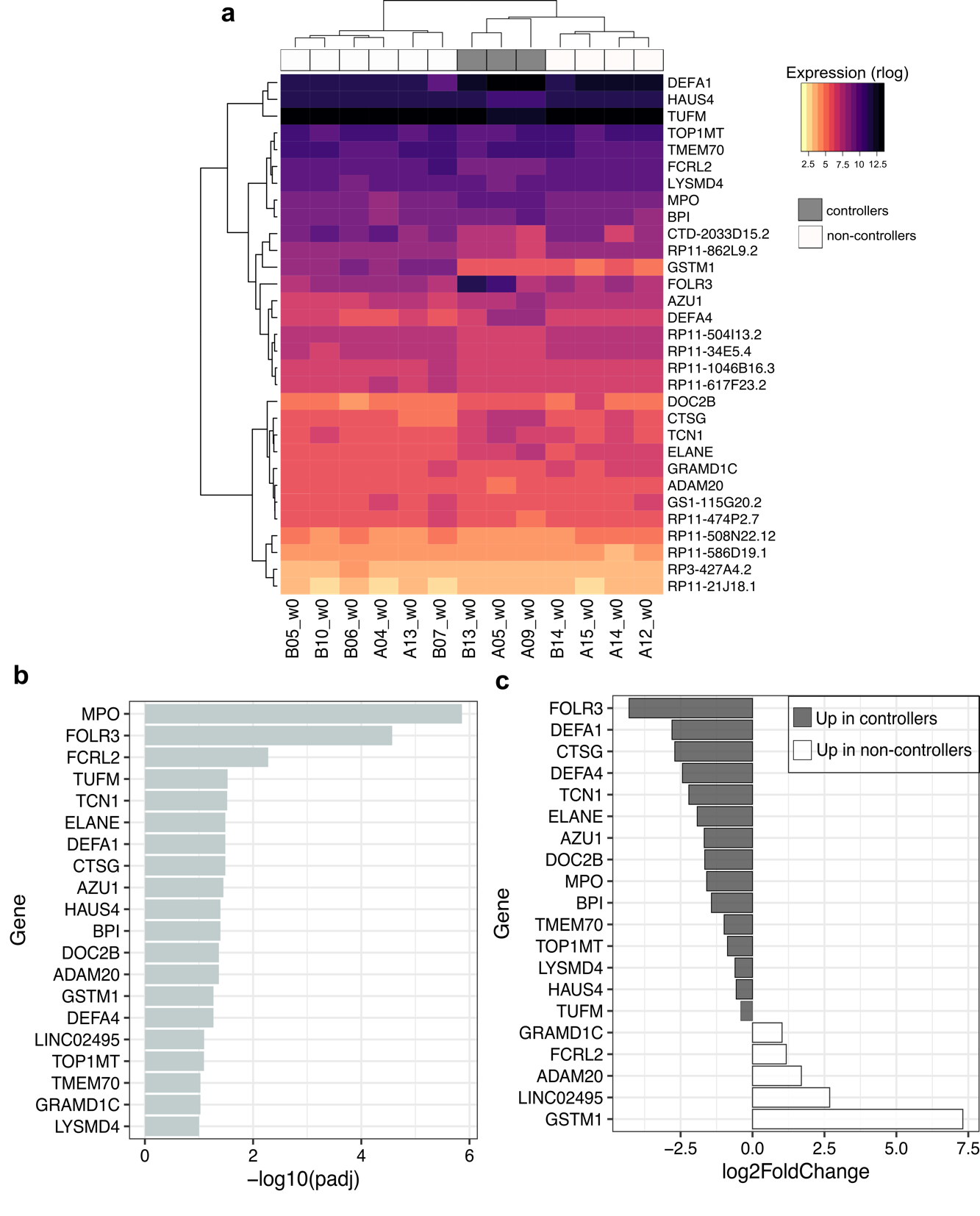
**

**Figure S14. Differentially expressed PBMC host genes between controllers and non-controllers at baseline. a**, Heatmap representation of DEG (adjusted *p*-value <0.1 and log2FoldChange = 0). Gene and sample-wise hierarchical clustering was performed on individual normalized read counts (rlog). The input matrix was scaled on rows to visualize changes in expression on gene level and columns to display relatedness of samples. **c-d**, Barplots showing DEG (annotated transcripts) sorted by log10 (adjusted p-value) (**c**) and log2 (fold change) (**d**). In plot **d**, gray and white bars represent upregulated genes in controllers and non-controllers, respectively.


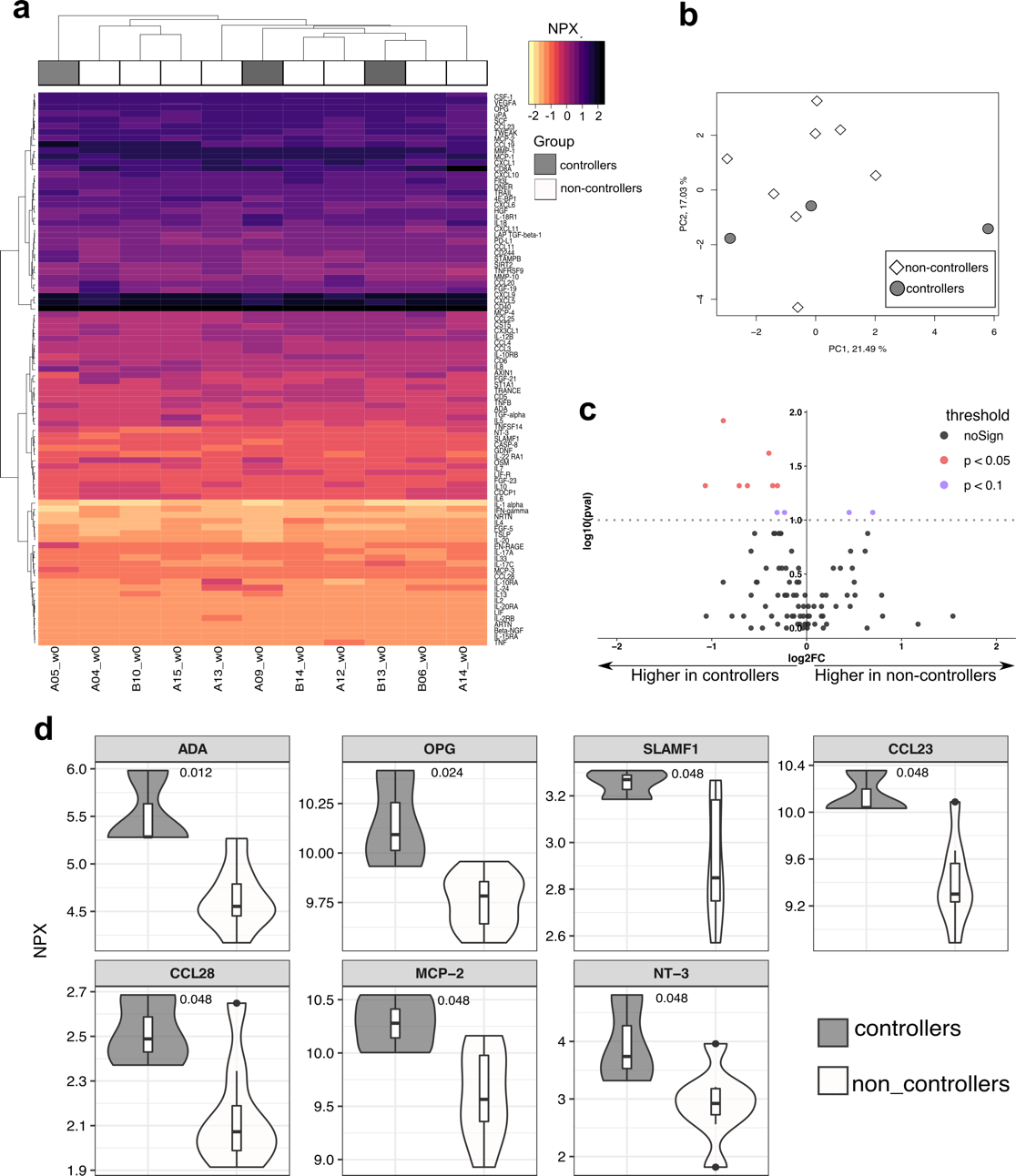


**Figure S15. Protein inflammation markers from controllers and non-controllers at baseline.** **a**, Heatmap of plasma protein abundance based on the full Olink-inflammation panel consisting of 92 assessed proteins. Unit variance scaling was applied to NPX levels. **b**, Principal component analysis based on the full Olink-inflammation panel in controllers (gray) and non-controllers (white). The plot shows the first two principal components and their relative contribution to overall variance. **c**, Volcano plots showing differentially-expressed proteins at p-value < 0.05 (red dots), p-value < 0.1 (violet dots) and not significantly different features (black dots). Y-axis displaying the p-value and x-axis showing the fold-change in logarithmic scale. **d**, Comparative analysis of significantly different proteins (p < 0.05) between controllers and non-controllers. Y-axis indicates NPX levels for each protein.

**
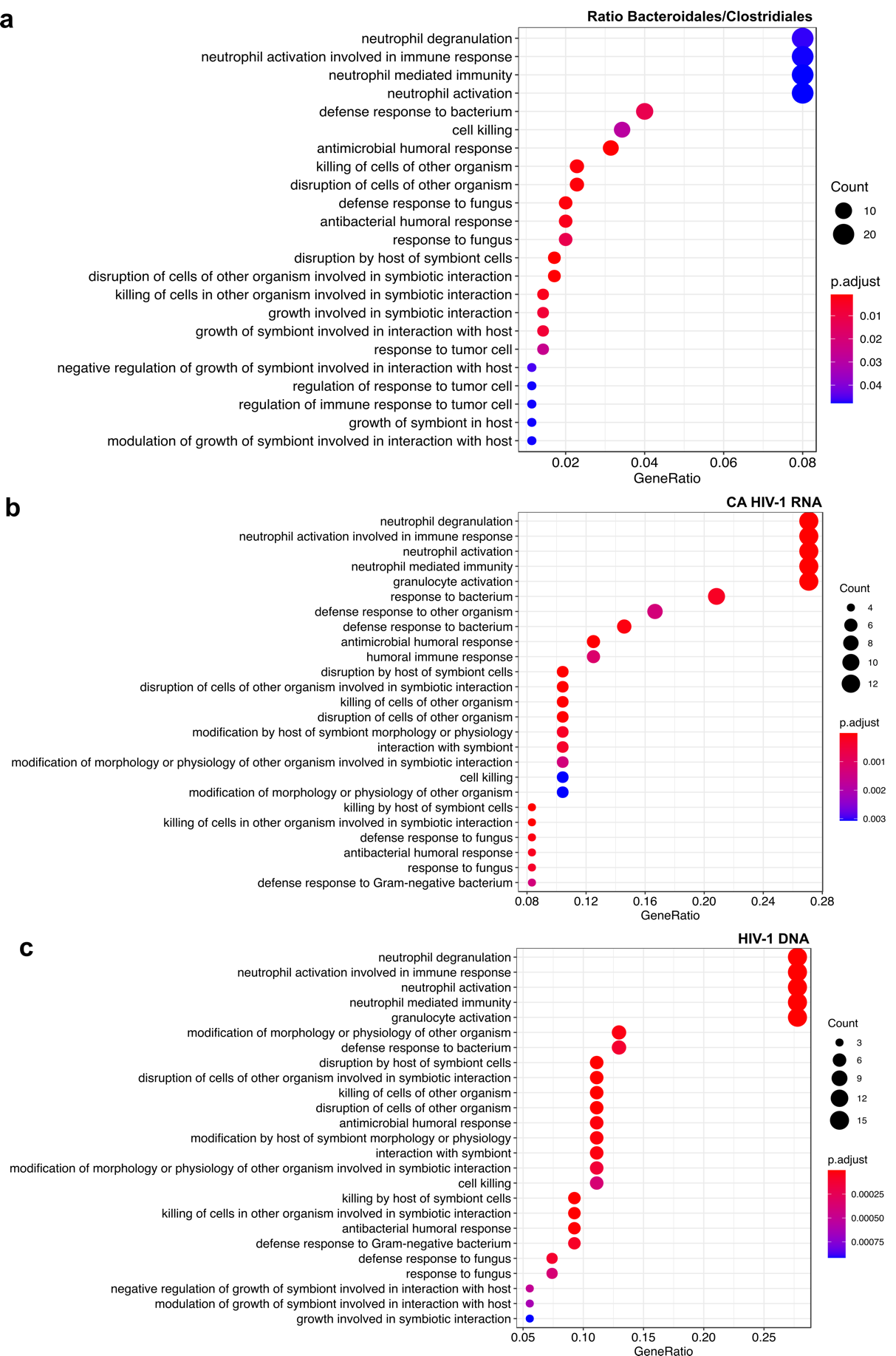
**

**Figure S16. Functional enrichment of transcripts correlated with the ratio *Bacteroidales:Clostridiales* and viral reservoir.** GO enrichment of transcripts significantly correlated (*q*-value ≤ 0.05) with **a**, ratio *Bacteroidales:Clostridiales*, **b**, CA HIV-1 RNA and **c**, HIV-1 DNA at study entry (pre-Vax) assessed by clusterProfiler. The x-axis reports the relative abundance of annotated transcripts in a given GO term, expressed as GeneRatio. Color scales indicate different thresholds of Bonferroni-adjusted *p*-values, and dots’ size represents the gene count of each functional GO cluster. Significantly enriched GO terms, number of genes associated to each term and adjusted *p*-values are provided in Additional file 3: Tables S5 and S6.

**
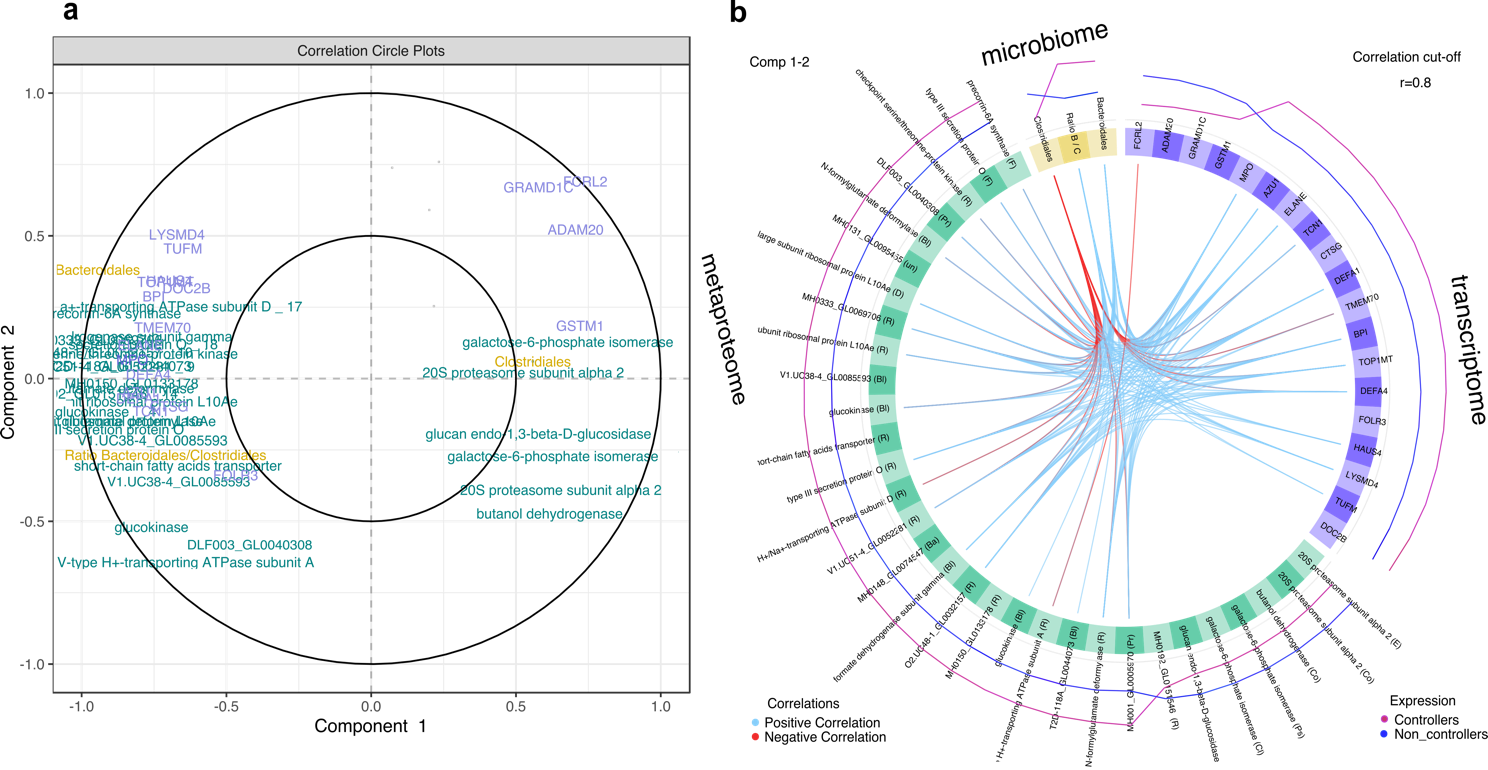
**

**Figure S17. Integrated analysis of microbiome, metaproteome and transcriptome data.** Feature correlation analysis between microbial biomarkers (relative abundance), differential bacterial proteins (*p*-value ≤ 0.025) and host annotated transcripts (adjusted *p*-value ≤ 0.1 and log2 fold change = 0). **a,** Variable plot displaying variables from each ‘-omic’ block, selected on component 1 and 2. Variable names from microbiome, metaproteome and transcriptome data are indicated in yellow, green and blue, respectively. Cluster of features indicate strong correlation between variables. **b**, Circos plot representing correlations between variables of each ‘-omic’ block. The correlation cut-off was set at 0.8. Inner light blue and red lines correspond to positive and negative correlation between connected features, respectively. Outer purple and dark blue lines indicate the variation of each features in viremic controllers and non-controllers, respectively. Protein-associated bacterial genera are reported in parentheses. Abbreviations: Bl; *Blautia*, R; *Ruminococcus*, Pr; *Prevotella*, Ps; *Pseudoflavonifactor*, Co; *Coprococcus*, D; *Dorea*, F; *Faecalibacterium*, Ba; *Bacteroides*, E; *Eubacterium*, un; undistinguishable, Cl; *Clostridium*.

**The BCN02 Study Group.** IrsiCaixa AIDS Research Institute-HIVACAT Hospital Universitari Germans Trias i Pujol, Badalona, Spain: Susana Benet, Christian Brander, Samandhy Cedeño, Bonaventura Clotet, Pep Coll, Anuska Llano, Javier Martinez-Picado, Marta Marszalek, Sara Morón-López, Beatriz Mothe, Roger Paredes, Maria C. Puertas, Miriam Rosás-Umbert, Marta Ruiz-Riol. Fundació Lluita contra la Sida, Infectious Diseases Department, Hospital Universitari Germans Trias i Pujol, Badalona, Spain: Roser Escrig, Silvia Gel, Miriam López, Cristina Miranda, José Moltó, Jose Muñoz, Nuria Perez-Alvarez, Jordi Puig, Boris Revollo, Jessica Toro. Germans Trias i Pujol Research Institute, Badalona, Spain: Ana María Barriocanal, Cristina Perez-Reche. Clinical Pharmacology Unit, Hospital Universitari Germans Trias i Pujol, Badalona, Spain: Magí Farré. Pharmacokinetic/pharmacodynamic modeling and simultation, Institut de Recerca de l’Hospital de la Santa Creu i Sant Pau-IIB Sant Pau, Barcelona, Spain: Marta Valle. Hospital Clinic- HIVACAT, IDIBAPS, University of Barcelona, Barcelona, Spain: Christian Manzardo, Juan Ambrosioni, Irene Ruiz, Cristina Rovira, Carmen Hurtado, Carmen Ligero, Emma Fernández, Sonsoles Sánchez-Palomino, and Jose M. Miró. Projecte dels NOMS-Hispanosida, BCN Checkpoint, Barcelona, Spain: Antonio Carrillo, Michael Meulbroek, Ferran Pujol and Jorge Saz. The Jenner Institute, The Nuffield Department of Medicine, University of Oxford, UK: Nicola Borthwick, Alison Crook, Edmund G. Wee and Tomáš Hanke.
